# Agarose-Based 3D Invasion Assay for Simultaneous Quantification of Tumor Cell Invasion and Extracellular Matrix Degradation

**DOI:** 10.3390/mps9040112

**Published:** 2026-07-28

**Authors:** Andreas R. Thomsen, Pascaline Kouam-Daniel, Bettina Priesch-Grzeszkowiak, Anja Grillenberger, Sandra Kumbruch, Ali H. Acikelli, Helmut Bühler, Christian Baues

**Affiliations:** 1 Department of Radiation Oncology, University of Freiburg, 79106 Freiburg, Germany; andreas.thomsen@uniklinik-freiburg.de; 2 Institute for Molecular Oncology, Radio-Biology and Experimental Radiotherapy, Marien Hospital Herne, University Clinic of Ruhr-University Bochum, 44265 Herne, Germany; bettina.priesch@elisabethgruppe.de (B.P.-G.); anja.grillenberger@elisabethgruppe.de (A.G.); sandra.kumbruch@elisabethgruppe.de (S.K.); helmut.buehler@rub.de (H.B.); christian.baues@elisabethgruppe.de (C.B.); 3 Department of Haematology and Oncology, Institute of Molecular Oncology and Experimental Therapeutics, Marien Hospital Herne, University Clinic of Ruhr-University Bochum, 44265 Herne, Germany; alihaydar.acikelli@elisabethgruppe.de; 4 Department of Radiotherapy and Radio-Oncology, Marien Hospital Herne, University Clinic of Ruhr-University Bochum, 44265 Herne, Germany

**Keywords:** 3D invasion assay, tumor invasion, matrix degradation, extracellular matrix, agarose

## Abstract

Tumor cell invasion is a critical step in local tumor progression, recurrence, and metastasis. Conventional two-dimensional migration assays and many existing three-dimensional invasion models often assess cell migration, invasion into the extracellular matrix and matrix degradation as separate endpoints, although these processes are tightly coupled in vivo. Therefore, robust and reproducible in vitro models are needed to investigate tumor cell invasion under defined extracellular matrix conditions. We developed an agarose-based three-dimensional invasion assay, termed the Freiburg 3D invasion assay, for the simultaneous analysis of tumor cell migration, invasion, and extracellular matrix degradation. The system consists of a 2.8% agarose matrix containing defined microcavities connected by a common loading channel. Tumor cells are seeded into these microcavities, where they form compact cell aggregates. The cavities are subsequently filled with collagen type I or extracellular matrix gel. After polymerization, the matrix-containing agarose strips are transferred into parking pockets, cultured for several days, and monitored by microscopy. Invasion distance, single-cell migration, and ECM-cleared area are quantified from serial microscopic images using image analysis software. The system distinguished weakly invasive MCF7 breast cancer cells from highly invasive MDA-MB-231 cells. In addition, treatment with a protease inhibitor and irradiation reduced tumor cell invasion and extracellular matrix remodeling, demonstrating the suitability of the assay for pharmacological and radiation-response studies. The Freiburg 3D invasion assay provides a practical and reproducible three-dimensional in vitro model for analyzing tumor cell invasion and protease-associated extracellular matrix degradation.

## 1. Introduction

The ability of tumor cells to form local recurrences and metastases depends, among other factors, on their invasive and migratory potential. Highly migratory or invasive tumor cells often display features of epithelial–mesenchymal transition (EMT), a process that substantially alters cell morphology and gene expression patterns [1,2]. To invade adjacent tissue, EMT-like tumor cells must overcome the extracellular matrix (ECM) and the basement membrane [3,4,5]. During tumor invasion, large amounts of proteases, including ADAMs, matrix metalloproteinases (MMPs), cathepsins, and urokinase-type plasminogen activator (uPA), are secreted by stromal cells, macrophages, mast cells, fibroblasts, and tumor cells themselves. These proteases contribute to ECM degradation and remodeling and thereby support tumor invasion and progression [6]. These biochemical processes are closely linked to the physical properties of the ECM, as recent 3D spheroid studies have shown that matrix stiffness affects tumor cell invasion, collagen remodeling and the propagation of tumor-induced mechanical stress into the surrounding ECM [7].

Because cell–cell and cell–ECM interactions play central roles in migration and invasion, there remains a need for suitable cell biological methods for the in vitro analysis of these processes under conditions that more closely approximate physiological tissue environments. Several 3D invasion models have been developed to overcome the limitations of conventional 2D migration and Transwell-based assays. For example, Aslan et al. described a 3D Matrigel drop invasion assay in which tumor cells are directly mixed with Matrigel, cultured as a drop, and monitored over several days to assess migration and invasion [8]. Fluorescence-based degradation assays using dye-quenched substrates, such as DQ-collagen I or DQ-collagen IV have also been established to visualize protease-dependent ECM/collagen degradation in living tumor cells and 3D matrix cultures. Upon proteolytic cleavage of the quenched substrate, fluorescence is released, generating a signal that is proportional to proteolytic activity and can be quantified by fluorescence or confocal microscopy [9,10,11,12]. A related DQ-BSA-based approach was later applied by Goertzen et al. in a 3D spheroid invasion model [13]. More recently, Den Daas et al. introduced 3D collagen models with defined preformed structures, such as clefts, interfaces, and channels, to analyze directed cell migration within collagen matrices [14].

These approaches represent important methodological advances and are more physiologically relevant than simple 2D assays. However, depending on the specific system, they may require spheroid generation, fluorogenic substrates, fluorescence or confocal microscopy, cell or nuclear labeling for normalization, dedicated image analysis or may still be affected by variability in spheroid size, initial cell positioning and matrix distribution.

In this work, we present the Freiburg 3D invasion assay, which complements these approaches by using defined agarose microcavities that provide a reproducible starting position for tumor cells and a common loading channel that standardizes the sequential introduction of cells and ECM or collagen. This design enables longitudinal bright-field-based quantification of tumor cell invasion under defined extracellular matrix conditions. In addition, the assay allows parallel analysis of cell aggregate growth and cell-mediated ECM clearing/remodeling within the same 3D compartment. Although the assay does not directly measure protease activity, the defined geometry of the matrix-filled microcavities allows ECM remodeling to be quantified and provides a standardized method that can be combined with DQ-based approaches in future applications to validate protease-dependent matrix degradation.

For the implementation of the assay, we developed an agarose-based matrix carrier, referred to as the Gel carrier, which is used for the three-dimensional culture and defined positioning of tumor cells and ECM components.

## 2. Experimental Design

### 2.1. Assay Principle

**Figure 1:**
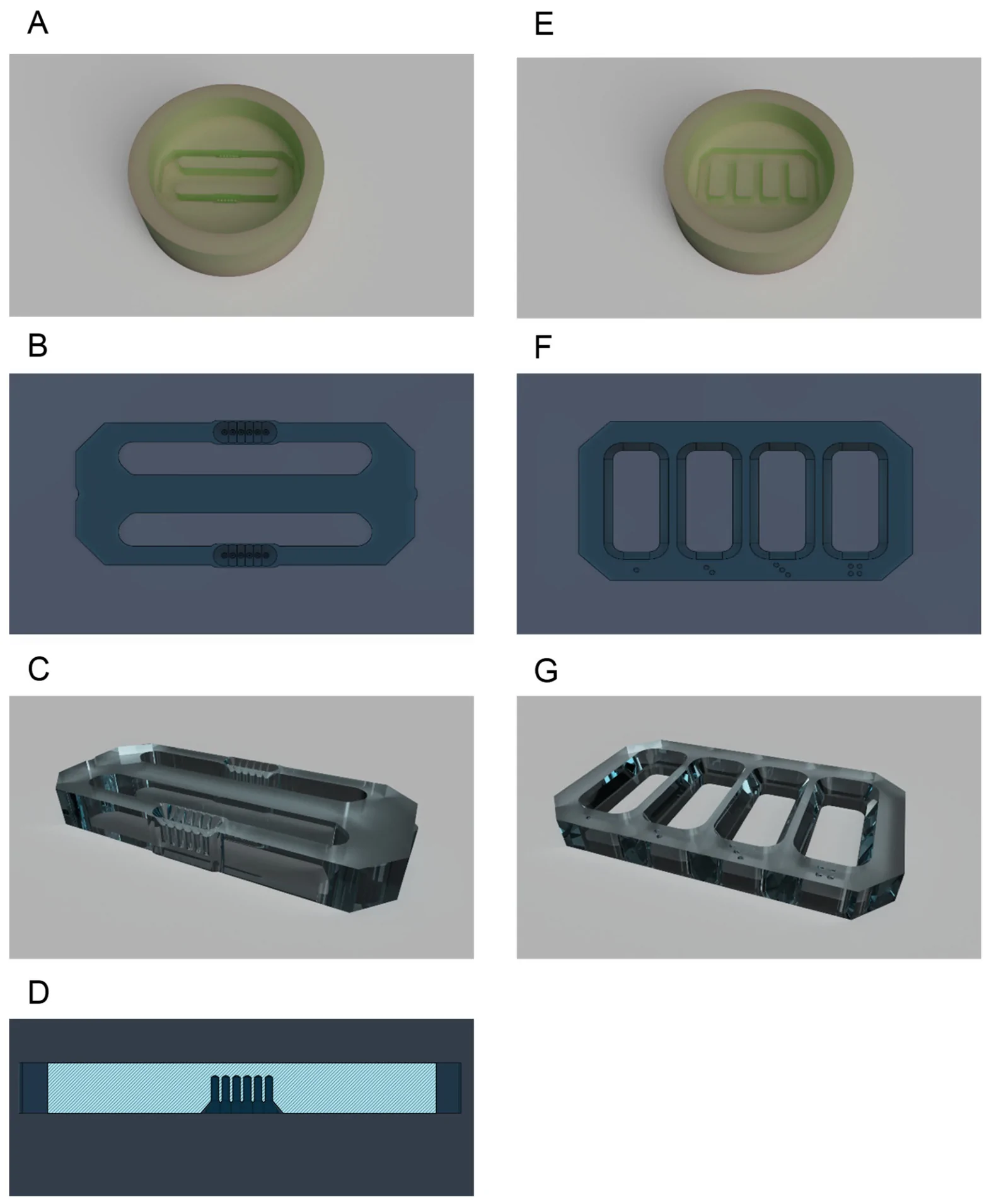
Technical illustrations and technical schematics: (**A**) Gel carrier silicone mold render. Serves as the negative mold for producing the agarose gel carriers; (**B**) Gel carrier top-view design. Represents the positive cell culture element with defined cavities; (**C**) Gel carrier render; (**D**) Gel carrier section design showing the common loading channel with six cavities; (**E**) Parking pocket silicone mold render; (**F**) Parking pocket top-view design; (**G**) Parking pocket render. Technical schematics is provided in the Supplementary Sections (Figure S1).

In this study, silicone casting molds were produced and used as reusable negative molds to generate two agarose-based components of the assay (Figure 1A,E): the Gel carriers (Figure 1B,C) and the parking pockets (Figure 1F,G). The Gel carrier is a 2.8% agarose-based culture carrier containing defined microcavities and a common loading channel (Figure 1D) through which tumor cells and extracellular matrix components, such as collagen type I or ECM gel, are introduced sequentially. The parking pockets are separate 2.8% agarose-based support structures produced using dedicated silicone casting molds; they stabilize the matrix-containing agarose strips during culture, handling, transport, and microscopic imaging (Figure 1F,G). Technical drawings and detailed dimensional specifications of these components are provided in the Supplementary Materials (Figure S1). The technical drawings of master molds for silicone casting molds are also provided in the Supplementary Sections (Figure S2).

The complete workflow, including the fabrication of both agarose components, cell seeding, matrix loading, long-term culture, imaging, and quantitative analysis, is referred to as the Freiburg 3D invasion assay.

### 2.2. Preparation of Agarose-Based Components

#### 2.2.1. Materials and Equipment

- Silicone casting molds: The Silicone molds were produced using a PTFE (Polytetrafluoroethylene) master mold and using the Dragon Skin 10 NV/1 Silicone (Cat. No. 09301-005-000181; KauPo, Spaichingen, Germany) according to the manufacturer’s instructions. Only defect-free silicone casting molds with intact loading channels and intact needle-like cavity-forming structures were used. These structures were inspected visually and their length and diameter were checked with a digital caliper.
- Agarose (Cat. No. 11404-03; SERVA, Heidelberg, Germany);
- Heated magnetic stirrer (IKAMAG RET, IKA, Staufen, Germany);
- Microwave;
- Paraffin embedding station with cooling plate (AP280-1, MICROM, Walldorf, Germany);
- Glass plates (3.7 cm Ø) with mounting screws;
- Weights (nuts and washers);
- Spatula;
- Disposable plastic Pasteur pipettes;
- Lint-free wipes;
- Sample cup, each containing 50 mL sterile standard Dulbecco phosphate-buffered saline (PBS) without Ca^2+^/Mg^2+^ (Cat. No. P04-361000; PAN-Biotech, Aidenbach, Germany);
- UV lamp (XX-20S Bench Lamp, 254 nm; 20 W, 230 V, 610 mm, Cat. No. UVPA95-0045-08, Analytik Jena, Jena, Germany).

#### 2.2.2. Preparation of the Agarose Solution and Pouring into Silicone Molds

The appropriate amount of agarose powder is weighed to prepare a 2.8% agarose solution and transferred into a Schott bottle containing a magnetic stir bar. The bottle is placed on a magnetic stirrer, and the corresponding volume of autoclaved distilled water is added rapidly while stirring. The suspension should be stirred for at least 5 min, ensuring that no large clumps remain. The solution is then heated in a microwave until it briefly boils and becomes completely clear and homogeneous. The agarose solution is subsequently autoclaved and stored until use.

**Figure 2:**
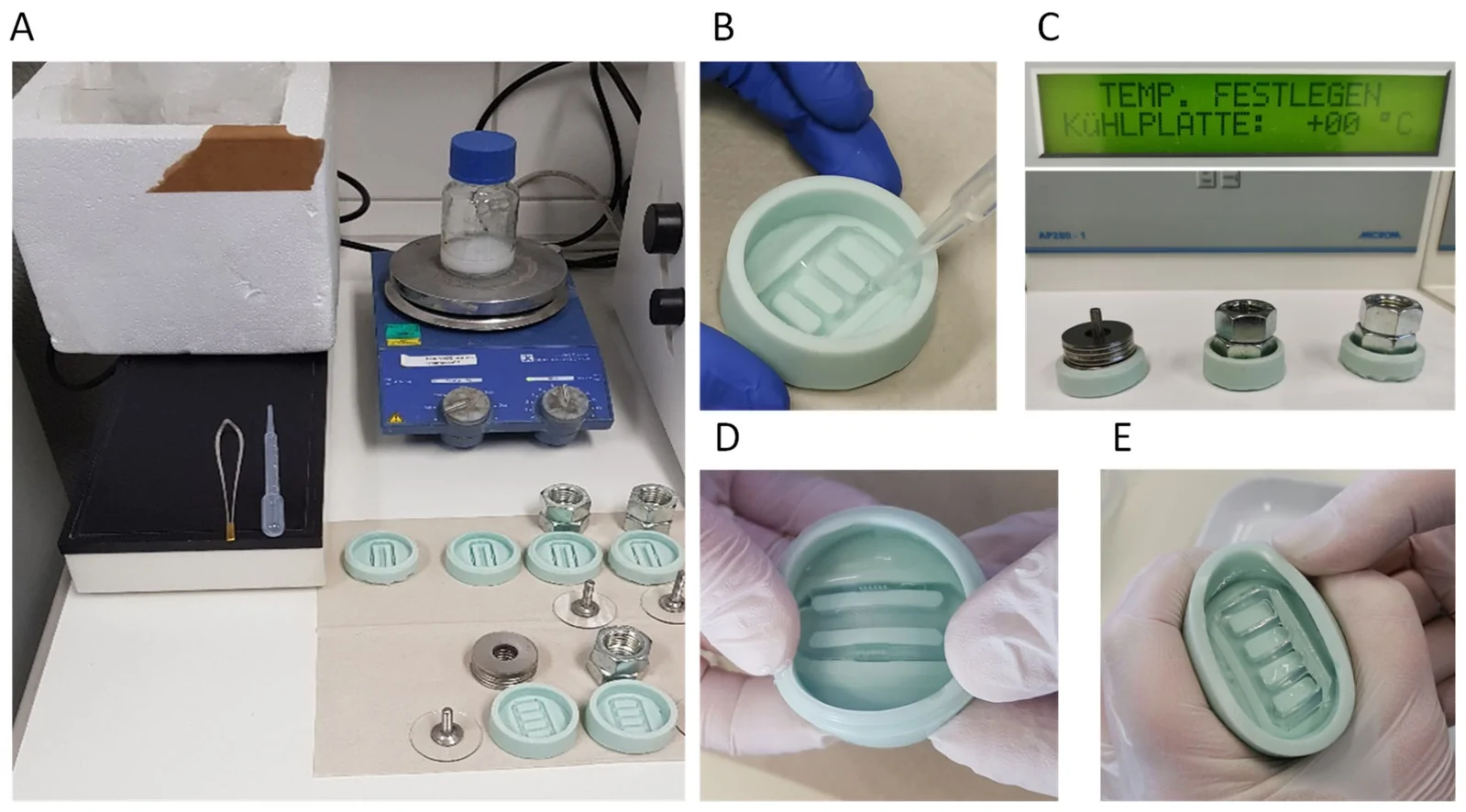
Fabrication of the agarose-based assay components: Gel carriers and parking pockets. (**A**) Materials: 2.8% agarose solution on a heated magnetic stirrer, spatula, disposable plastic Pasteur pipettes, six silicone molds (four molds as negative molds for producing Gel carriers and two molds as negative molds for producing parking pockets), three glass plates with mounting screws, and weights (six nuts and four washers). (**B**) Pouring of the agarose solution into the molds, shown here using the silicone mold for the parking pockets as an example. (**C**) Covering of the agarose solution with the glass plate and cooling on the cooling plate with retaining screws for weighting. (**D**) Removal of the Gel carrier from the silicone mold. (**E**) Removal of the parking pockets from the silicone mold or negative mold.

For the preparation of Gel carriers and parking pockets, 2 mL and 1.5 mL of agarose solution, respectively, are required; the solution is kept hot and liquid on the heated magnetic stirrer at 130–150 °C until casting is completed. The silicone molds are placed on the heated magnetic stirrer next to the Schott bottle containing the agarose solution to pre-warm them. Using a Pasteur pipette, the corresponding volume of agarose solution is aspirated and rapidly pipetted into the molds so that they are completely filled but not overfilled. Immediately afterwards, a spatula is gently moved over the regions containing pockets or cavities to remove air bubbles. The glass plate is then slid into the silicone mold from one side to the other to cover the agarose solution. The molds are subsequently placed on the cooling plate and weighted down with retaining screws for 10–15 min (Figure 2A–C).

**Figure 3:**
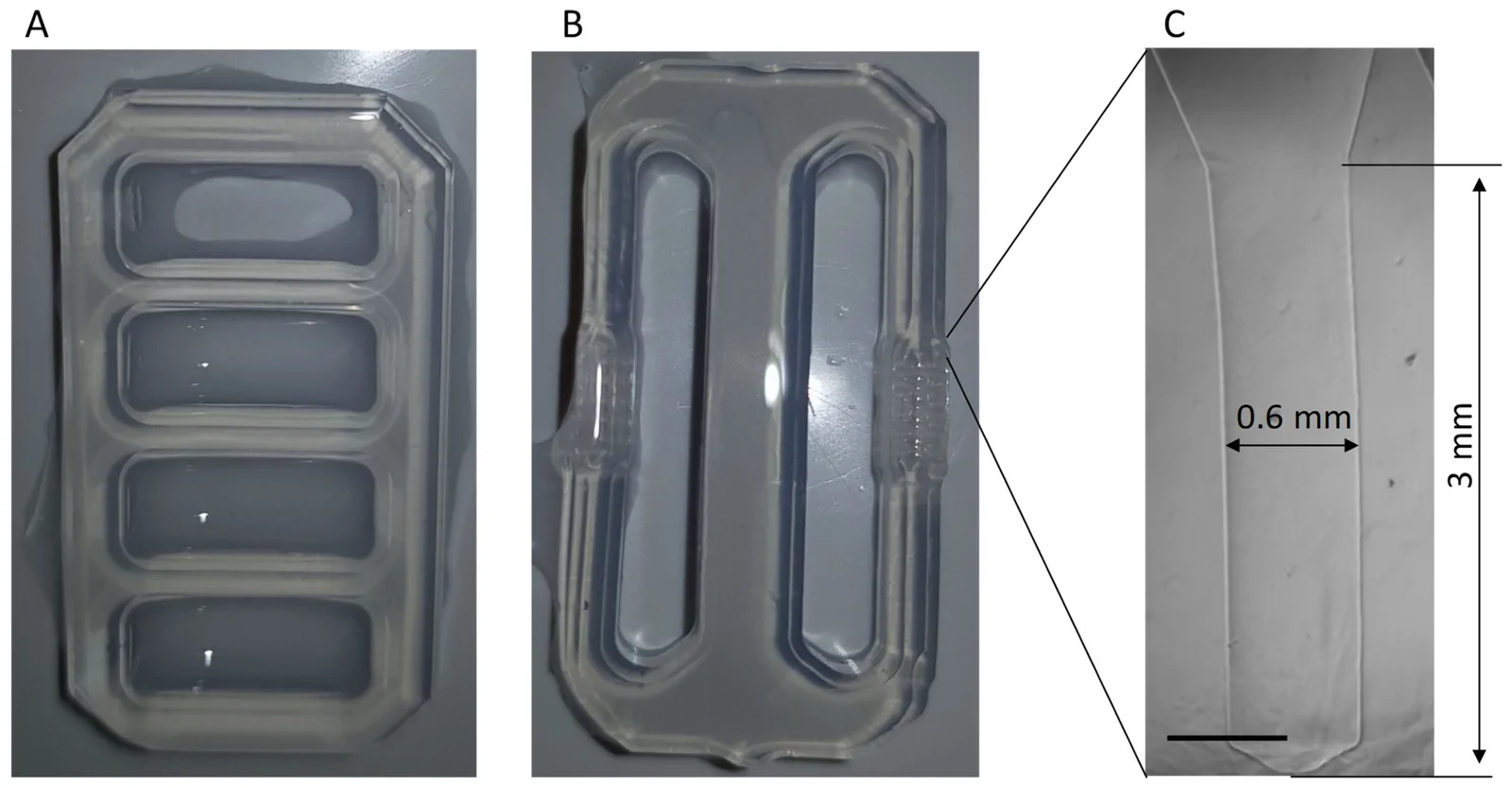
Representative quality-controlled Gel carriers and parking pockets. Both components consist of 2.8% agarose and measure approximately 28 × 12 mm. (**A**) Agarose parking pocket with four parking positions; this component serves as a support structure for the matrix strips during culture, transport, and microscopy. (**B**) Gel carrier for cell culture. The carrier contains a central bridge for handling with forceps and one strip on each side, each containing six cavities or pockets in which cells and subsequently ECM/collagen are combined. Each strip contains a common channel through which cells and then ECM are loaded so that they enter the cavities. (**C**) Microscopic image of one of the six cavities of the common channel. The conical cavities or pockets have a diameter of 0.6 mm and are approximately 3 mm deep. The common channel has a volume of 15 µL. (Scale bar = 0.5 mm).

After polymerization or solidification of the agarose, the Gel carriers and parking pockets are carefully removed from the silicone molds. This step requires careful handling and manual precision. First, the edges of the agarose gel are loosened by gently moving the mold and applying lateral pressure. Excessive stretching of the silicone molds should be avoided; instead, even pressure should be applied (Figure 2D,E). The released Gel carriers and parking pockets are then inspected for cracks, breaks, air bubbles, and artifacts (Section 2.6). Components that pass this quality control (Figure 3) are stored in PBS until further use.

Before use in the 3D invasion assay, the Gel carriers and parking pockets are subjected to an additional UV decontamination step in a cell culture hood. For this purpose, the open containers holding the components are placed in a box lined with aluminum foil and irradiated with a UV lamp (20 W, 254 nm, 230 V) for 20 min. This UV exposure time was selected during protocol development from tested exposure time from 10 to 30 min. To verify sterility, a representative subset of matrices from each production batch is incubated in culture dishes containing standard cell culture medium supplemented with either 2.5% or 10% fetal calf serum (FCS) (Cat. No. P40-37500; PAN-Biotech, Aidenbach, Germany). Incubation is performed for 21 days at 37 °C and 5% CO_2_. This incubation time was chosen based on the observation that our less invasive cell line requires at least 14 days to invade the matrix gel. After successful sterility testing, sterile agarose gel are subsequently stored in PBS without Ca^2+^/Mg^2+^ at room temperature for up to six months.

### 2.3. Freiburg 3D Invasion Assay

#### 2.3.1. Materials and Equipment

**Figure 4:**
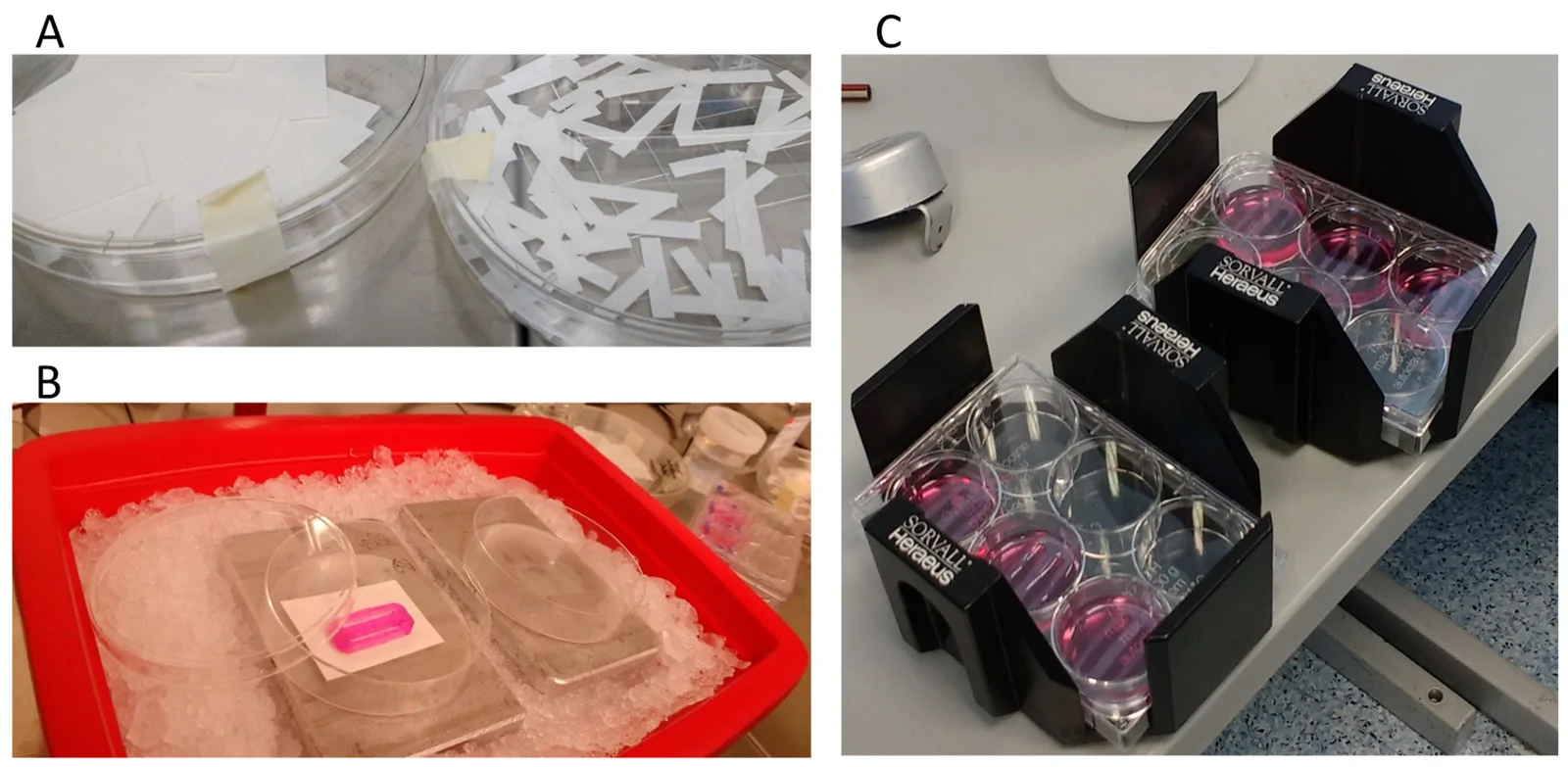
(**A**) Chromatography paper strips (5.5 mm × 3 cm) and rectangles (3.5 cm × 4.5 cm) used to remove liquid from channels and cavities. (**B**) Ice box with cooling plate, PBS, ethanol in a 50 mL bottle, 6-well plate containing medium and Gel carriers, chromatography paper in a 10 cm dish, and a cup containing Gel carriers in PBS. The process of drawing liquid from the matrix is shown, allowing cells or ECM to descend into the cavities of the channel. (**C**) Arrangement of the plate for centrifugation, either to sediment cells into the cavities or to remove air bubbles from the cavities.

- Sterile Gel carriers (Section 2.2.2).
- Sterile parking pockets (Section 2.2.2).
- Forceps; handle the Gel carriers and parking pockets carefully because they may crumble or break.
- Small scissors or a scalpel.
- 70% ethanol in a 50 mL bottle for disinfecting the working surface, scissors, and forceps.
- DPBS without Ca^2+^ and Mg^2+^ (Cat. No. P04-361000; PAN-Biotech, Aidenbach, Germany).
- Medium containing 1% penicillin/streptomycin (P/S) (Cat. No. P06-07100; PAN-Biotech, Aidenbach, Germany) and 2.5% FCS for Gel carrier and parking pocket equilibration and cell culture during the assay.
- Three 6-well plates (Sarstedt, Nümbrecht, Germany) for culture.
- Compresses/wipes; scissors and forceps should be dabbed dry after disinfection to remove residual ethanol completely.
- Chromatography paper (Cat. No. 3030672; Whatman, Little Chalfont, UK): cut into strips (5.5 mm × 3 cm) and rectangles (3.5 cm × 4.5 cm) (Figure 4A), then sterilised by autoclave. The strips are used to remove liquid from the channel; the rectangles are used to draw liquid from the Gel carrier and thereby support the descent of cells or ECM into the cavities of the channel.
- 10 cm dishes (Sarstedt, Nümbrecht, Germany) for dabbing, loading, and cutting the strips of the Gel carrier.
- Cooling plates and ice boxes for cooling dishes and Gel carriers before loading ECM or collagen, and for keeping collagen or ECM cold to prevent premature polymerization (Figure 4B).
- Centrifuge (Sigma MTP Swing Out Rotor 11222/13222, Sigma, Osterode am Harz, Germany).
- Light microscope (Nikon Eclipse Ts2) and camera (Nikon DS-VI1); Nikon, Düsseldorf, Germany.
- NIS-Elements Basic Research software, version 4.60.00; Nikon, Düsseldorf, Germany.
- Collagen type I (Cat. No. 804592; Sigma-Aldrich, Taufkirchen, Germany).
- ECM gel (Cat. No. E1270; Sigma-Aldrich, Taufkirchen, Germany).
- GI254023X, an ADAM10/17 inhibitor (Cat. No. SML0789; Sigma-Aldrich, Taufkirchen, Germany).

#### 2.3.2. Cell Culture

The breast cancer cell lines MDA-MB-231 and MCF7 and the glioblastoma cell line U-251 MG were obtained from the American Type Culture Collection (ATCC, Manassas, VA, USA). MDA-MB-231 and U-251 MG cells were cultured in DMEM (P04-04510; PAN-Biotech, Aidenbach, Germany), whereas MCF7 cells were cultured in RPMI 1640 (P04-18525; PAN-Biotech, Aidenbach, Germany). Media were supplemented with 10% FCS and penicillin/streptomycin (10,000 U/mL and 10 mg/mL, respectively). Cells were maintained at 37 °C in a humidified atmosphere containing 5% CO_2_.

#### 2.3.3. Invasion Assay

Day 1: Preparation of the Gel carriers and cell seeding

(a) Equilibration of Gel Carriers with Culture Medium

Before use, the Gel carriers are first incubated in medium to remove PBS from the agarose gel. The matrices are transferred into a 6-well plate and covered with 4 mL of medium containing 2.5% FCS and 1% P/S. The plate is then centrifuged at 300× *g* for 1 min to remove air bubbles from the cavities of the channel (Figure 4C). Afterwards, the Gel carriers are incubated for 30 to 60 min at 37 °C and 5% CO_2_.

(b) Preparation of Cell

A cell suspension of MCF7 or MDA-MB-231 cells at 400,000 cells/mL is prepared in a 50 mL Falcon tube. For this purpose, cells that have been detached, for example by trypsinization, are counted and resuspended in medium containing 2.5% FCS and 1% P/S.

(c) Loading the Channel of the Gel Carrier with Cells

**Figure 5:**
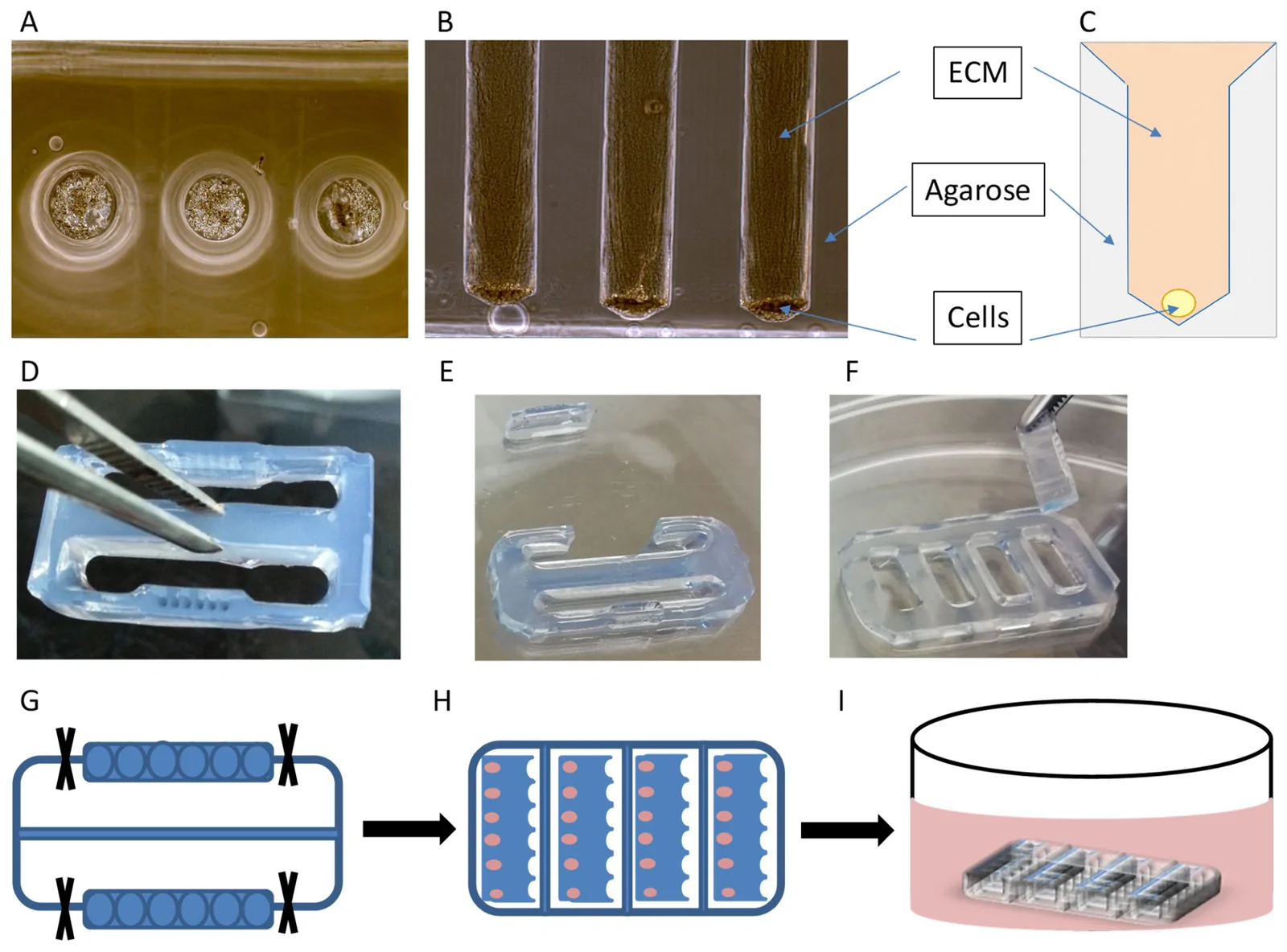
3D invasion assay. (**A**–**C**) Representation of cavities (three of six) in the common channel of one strip of the Gel carrier containing MDA-MB-231 cells and collagen type I. (**A**) Top-view image of cells (approximately 1000 cells per cavity) immediately after seeding, without collagen. (**B**) Image after addition of collagen solution into the cavity. (**C**) Schematic representation of a cavity filled with cells and extracellular matrix. In the invasion assay, cavities can either be filled with extracellular matrix or left without ECM as a control. (**D**,**G**) Frei Gel carrier grasped with forceps at the central bridge; one strip with six cavities or pockets is located on each side, and these are loaded through a common channel. (**E**,**G**) After loading with cells and ECM and after gel polymerization, both strips are separated from the central bridge. (**F**,**H**) The strips are positioned laterally or horizontally within the parking pockets. The parking pockets stabilize the strips during transport and microscopy. (**I**) For incubation and microscopic imaging, the parking pockets are placed in a 3.5 cm culture dish or in a 6-well plate.

Before cells are added to the channel (Figure 5A–C), the medium should be removed from the channel and from the six cavities. First, the Gel carrier is removed from the equilibration medium using forceps and placed upright in an empty 10 cm dish. To avoid breaking the central bridge of the Gel carrier, the carrier should be grasped with the forceps at the end of the central bridge (Figure 5D).

Using a chromatography paper strip (5.5 mm × 3 cm), the medium is drawn out of the channel. The strip is held with forceps at one end and inserted vertically into the channel to ensure uniform removal of medium. The process is continued until the medium has been absorbed. During liquid removal, care must be taken not to dry the channel or pockets completely, because this would promote air bubble formation during subsequent loading with cell suspension or ECM. Air bubbles would prevent the cells or ECM from reaching the cavities of the channel accurately after centrifugation.

After removal of medium from the common channel of the Gel carrier, cells are seeded as follows. The Gel carrier is placed on a rectangular piece of chromatography paper (3.5 cm × 4.5 cm) that has been placed in a new, dry 10 cm dish. Then, 15 µL of cell suspension (400,000 cells/mL) is carefully pipetted into the common channel. The carrier is left for approximately 5 min until the cells descend into the cavities of the channel due to the capillary action of the chromatography paper.

After loading the channel with cells, the Gel carriers are transferred into a 6-well plate containing 1 mL of medium. A centrifugation step is performed at 300× *g* for 2 min at room temperature to allow the cells to settle into the depth of the channel. Subsequently, the position of the cells in the cavities is checked microscopically (Figure 5A). The carriers are then incubated at 37 °C and 5% CO_2_ for 24 h.

*For loading the channel, the Gel carriers are placed on dry rectangular chromatography paper in a 10 cm dish. This enables cells or ECM to be drawn into the cavities of the channel. Centrifugation should be limited to the minimum force and duration required (300× g for 1–2 min) for bubble removal and cell positioning. Higher forces or longer centrifugation times should be avoided to prevent damage or breakage of the agarose gel carriers.*

Day 2: Addition of ECM or Collagen to the Gel Carriers

(a) Preparation of ECM or Collagen

*All steps are performed on ice because ECM and collagen polymerize rapidly at room temperature.*

Prepare an ice box or container filled with ice (Figure 4B); it should accommodate two cooling plates side by side. Thaw ECM on ice and prepare the collagen type I solution according to the manufacturer’s instructions and on ice as well. Chromatography paper strips and rectangles, 10 cm dishes, and 6-well plates are also prepared.

(b) Removal of Medium from the Common Channel of the Gel carrier

Two cooling plates are placed on ice, and a 10 cm Petri dish is placed on top to cool. The matrices containing cells from the previous day are placed upright in the cooled Petri dish and left there for 5 min so that the matrix also cools. This step is particularly important when using ECM. *To avoid unnecessary cold exposure, this step should be performed rapidly.* Using a chromatography paper strip, the liquid is removed from the common channel, but not completely*. If the cavities become completely free of liquid, air bubbles will form and the ECM cannot enter the cavities uniformly.*

(c) Loading ECM/Collagen into the Gel carriers: the “Elevator Down” Principle

After medium has been removed from the channels, 15 µL undiluted ECM gel or collagen type I is added dropwise into the common channel. The channel should be filled up to the rim. The Gel carrier is then placed on dry rectangular chromatography paper (Figure 4B). The carrier is left undisturbed until the ECM is drawn from the common channel into the cavities by the chromatography paper. Once the liquid level has decreased, additional ECM is immediately added to the common channel, and the process is repeated twice to ensure that all pockets are completely filled with ECM gel. The Gel carriers are then placed into a 3.5 cm culture dish or into a 6-well plate containing 1 mL medium and incubated at 37 °C and 5% CO_2_ for 30 to 60 min to allow ECM or collagen polymerization.

(d) Separation of the Strips from the Central Bridge and Storage in Parking pockets

**Figure 6:**
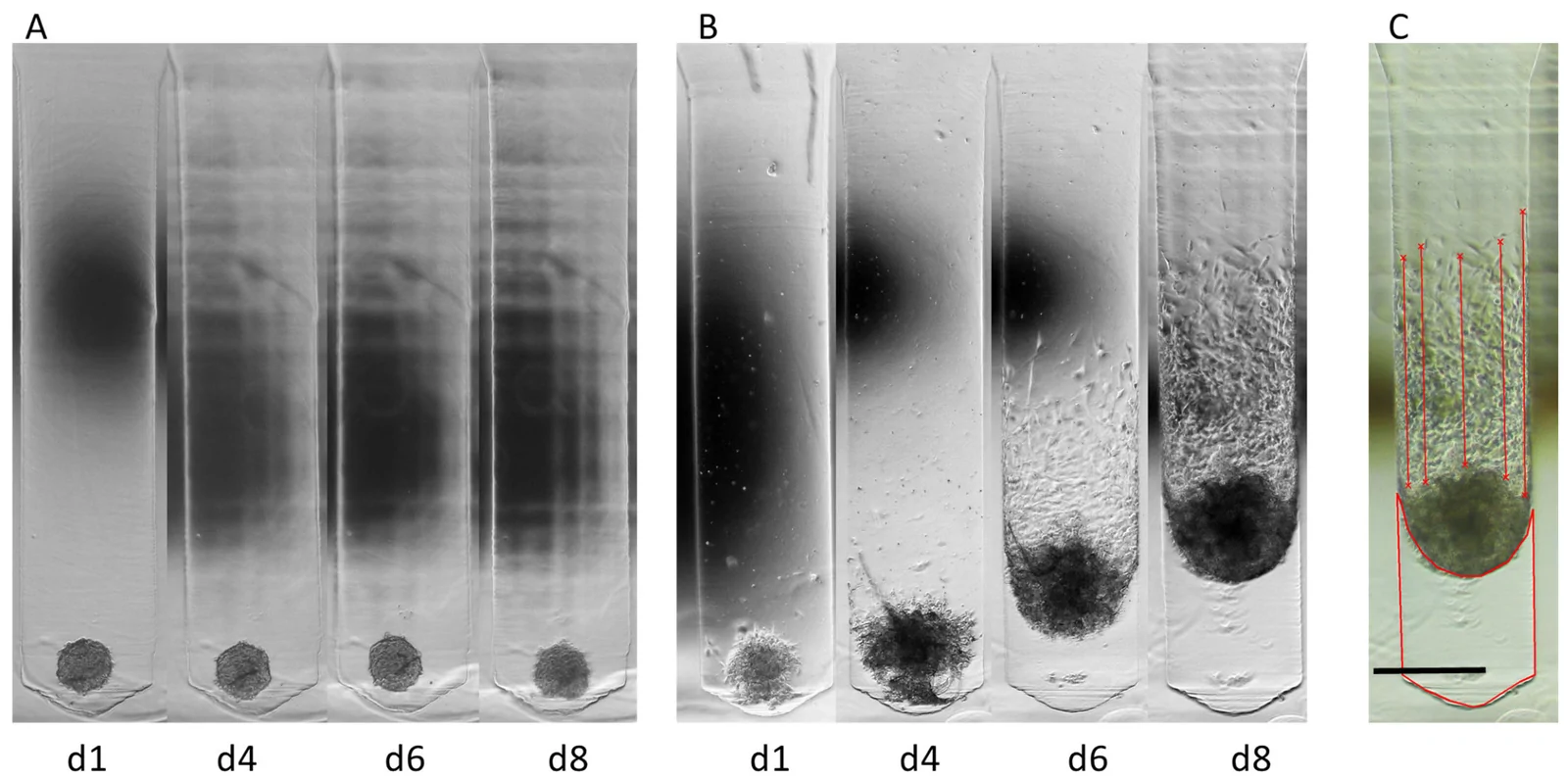
Measurement of invasion and ECM clearing/remodeling using NIS-Elements BR version 4.60.00. 6000 cells of the glioblastoma cell line U-251 MG were seeded into the agarose pockets of the Gel carrier, corresponding to approximately 1000 cells per cavity. The pockets were either filled with extracellular matrix (ECM) or cultured without ECM, so that cells were either covered with ECM or left without ECM as a control. Microscopic images of each pocket were acquired on day 1 (the day of ECM addition), day 4, day 6, and day 8. (**A**) Cells cultured in the absence of ECM. (**B**) Cells cultured in the presence of ECM. (**C**) Measurement of invasion: in each image, the five most extended cellular protrusions were manually marked (red lines), and the lengths were recorded in µm in an Excel table. To measure ECM clearing/remodeling, the cleared ECM area was manually marked in each image (red polygon). The areas were then recorded in µm^2^ in Excel and exported for further analysis. Cell aggregates size can be measured in the same way as ECM clearing by outlining the aggregates are in each image and the volume is then recorded and analysed. (Scale bar = 0.5 mm).

After polymerization of ECM or collagen, both strips containing the respective channels are carefully cut from the central bridge using scissors or a scalpel (Figure 5E,G). The strips are tilted outward or placed horizontally into the parking pockets to stabilize them during transport, culture, and microscopy in a 6-well plate (Figure 5F,H). Before this step, the parking pockets are equilibrated for 30 to 60 min in medium containing 2.5% FCS and 1% P/S in the incubator. The parking pockets containing the strips are overlaid with 1.5 mL of medium (Figure 5I). From this step onward, cells can be irradiated or treated with protease inhibitors, activators, or other desired substances. The first image acquisition is then performed. Cells can be monitored over several days or weeks by repeated imaging using a microscope, for example with a 4× or 10× objective (Nikon) (Figure 6A,B).

### 2.4. Measurement and Quantification of Invasion and ECM/Collagen Degradation

Using the acquired images, invasion distances or invasion’s length (µm), migration distances of individual cells (µm), and the area of cleared ECM/collagen (µm^2^) can be measured with NIS-Elements BR 4.60.00 software, similarly to the method described by Kim and colleagues (Figure 6) [15]. To measure invasion distance, five individual lines are drawn in the software under “Annotations and Measurements” and “Length”, starting from the point where cells detach from the spheroid or cell aggregate and ending at the farthest point to which they have migrated into ECM or collagen. Depending on the objective used for image acquisition, invasion distances are documented in µm in an Excel spreadsheet. To measure the area of cleared/degraded ECM or collagen, the region of interest is marked or outlined using the “Polygon” tool (Figure 6C). As for invasion distances, the measured cleared/degraded area is noted in µm^2^ in an Excel spreadsheet. The spreadsheet can then be exported for further calculations and analyses. The system also enables measurement of cell aggregate size in the absence (Figure 6A) or presence (Figure 6B) of ECM across a defined incubation time, allowing matrix-dependent effects on cell growth to be assessed. We observed that tumor cells grew markedly faster in the presence of ECM gel than without ECM gel. Moreover, cell growth differed depending on whether ECM gel or collagen type I was used (Figure S3).

### 2.5. Analysis of Invasion in the Breast Cancer Cell Lines MCF7 and MDA-MB-231

**Figure 7:**
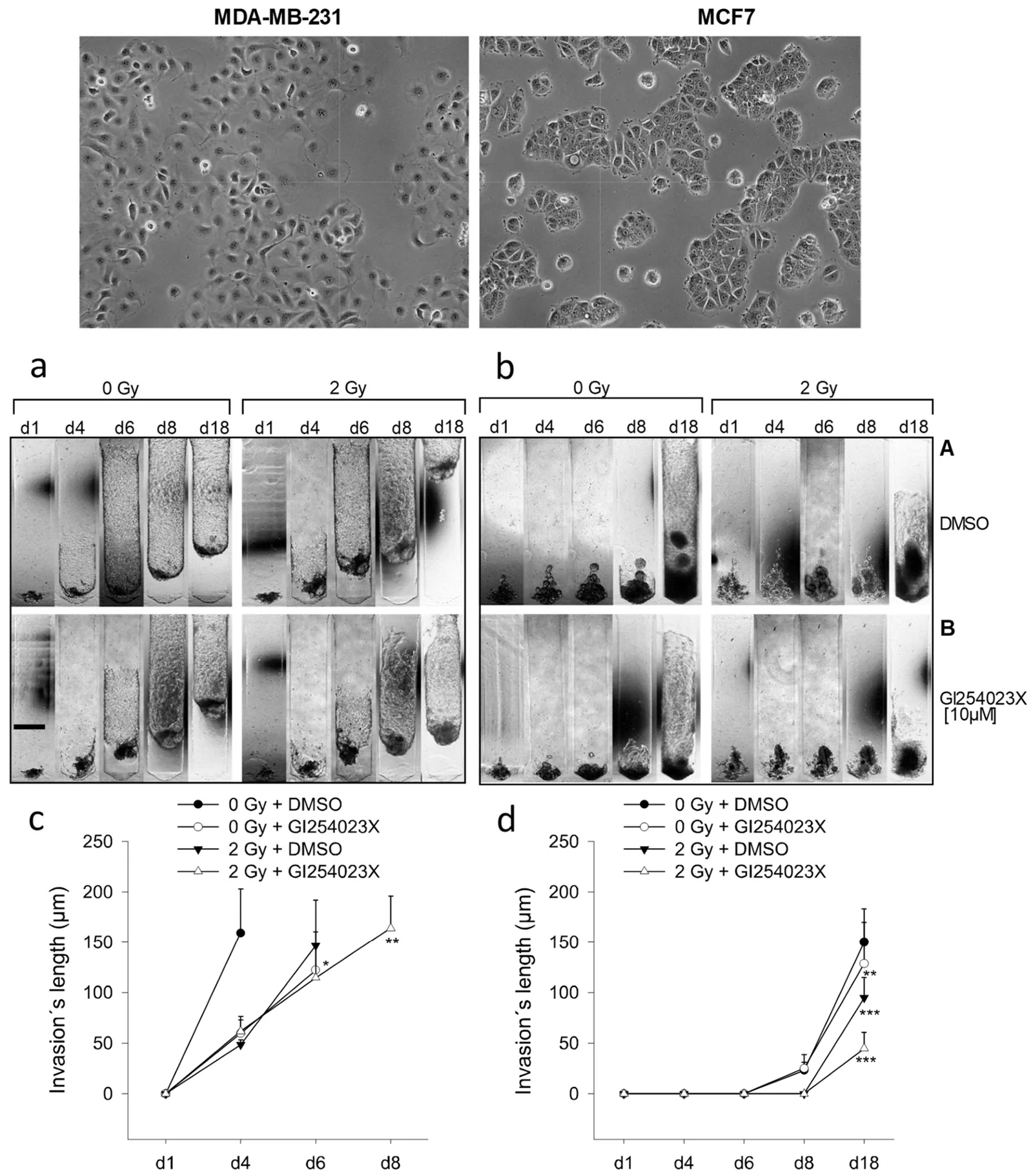
Invasion of breast cancer cells after irradiation and treatment with a protease inhibitor. Top: 2D growth of MDA-MB-231 and MCF7 cells. Images acquired 48 h after seeding (250,000 cells/3 cm culture dish) show morphological differences between the cell lines. (**a**) Growth of MDA-MB-231 and (**b**) MCF7 cells in ECM gel and inhibition of cell invasion by inhibition of the metalloproteinases ADAM10/17. Cells were irradiated with 2 Gy and treated with 10 µM GI254023X. Irradiation was performed approximately 2 h after ECM addition using a linear accelerator (Synergy S, Elekta, Hamburg, Germany) at a dose rate of 5 Gy/min at room temperature. GI254023X was added approximately 30 min before irradiation. (**c**,**d**) Quantification of invasion distance (µm) of (**c**) MDA-MB-231 and (**d**) MCF7 cells. Microscopic images of each pocket were acquired at several time points (d1, d4, d6, d8 and d18). Day 1 represents the day on which ECM gel was applied to the cells. Error bars indicate standard deviations. Statistical comparisons between irradiated or treated samples and the non-irradiated, untreated control (0 Gy + DMSO) were performed using Student’s *t*-test (GraphPad Prism version 8.2.1; GraphPad Software Inc., La Jolla, CA, USA). Data are presented as mean ± SD; * *p* < 0.05; ** *p* < 0.01; *** *p* < 0.001; *n* = 3 independent experiments. Per experiment and per condition or treatment, four Gel carriers were used: four carriers correspond to eight strips/channels and thus 48 cavities. (Scale bar = 0.5 mm).

The extracellular matrix is an important component of the tumor microenvironment and influences tumor cell migration and invasiveness [4]. In several tumor entities, proteases have been described as regulators of malignant disease progression. Using the Freiburg 3D invasion assay, we analyzed invasion and protease activity of breast cancer cell lines and correlated these findings with the properties of these cell lines described in the literature (Table 1). In our 3D invasion assay, MCF7 cells also showed markedly lower invasiveness and ECM remodeling than MDA-MB-231 cells (Figure 7), and the results obtained with our invasion assay therefore correlated with the information summarized in Table 1.

Based on our previous findings showing that ionizing radiation activates the metalloproteinase ADAM10 and thereby promotes transendothelial migration of tumor cells through VE-cadherin-dependent weakening of the endothelial barrier [16], we investigated whether ADAM10/17 inhibition also affects tumor cell invasion in a three-dimensional ECM environment. For this purpose, MDA-MB-231 and MCF7 cells were treated with GI254023X and/or irradiated with 2 Gy in the Freiburg 3D invasion assay. In the absence of the protease inhibitor, the MCF7 cell line required approximately three times longer (18 days) than the MDA-MB-231 cell line to fully colonize the ECM. During the growth of MDA-MB-231 cells, ECM-clearing occurred early, by day 4, whereas no ECM reduction was observed in MCF7 cells even after three weeks (Figure 7(aA,bA)). In both cell lines, treatment with the protease inhibitor slowed invasion (Figure 7(aB,bB),c,d). To determine whether reduced invasion could be explained by impaired cell viability, we analysed metabolic activity/Proliferation and clonogenic survival after GI254023X treatment. MTT assays performed 24, 48 and 72 h after treatment showed only minor effects at concentrations below 25 µM including the 10 µM concentration used in the invasion experiments. Clonogenic assays confirmed the expected radiation dose-dependent reduction in colony formation while the addition of 10 µM GI254023X caused only limited additional in both cell lines (Figure S4). Thus, reduced invasion after GI254023X treatment is unlikely to be explained solely by reduced cell viability.

**Table 1:** Molecular classification of the breast cancer cell lines MDA-MB-231 and MCF7.

Cell Line	Molecular Subtype	Tumorigenicity *	Reference
MDA-MB-231	Claudin-low or triple-negative	+++	[17]
MCF7	Luminal A	+	[18]

* [19,20]. + Non-invasive; without estrogen, these cells do not form tumors in nude mice. +++ Invasive; these cells form tumors in nude mice and metastasize.

### 2.6. Troubleshooting

Technical problems may occur during preparation and use of the Freiburg 3D invasion assay. Common problems, prossible causes and recommended solutions are summarized in Table 2. 

## 3. Discussion

Tumor cell invasion is a multistep process in which cell migration, cell–ECM adhesion, mechanical cell deformation, proteolytic matrix degradation, and adaptation to the physical properties of the extracellular matrix are closely coupled. In native tissues, tumor cells do not migrate on two-dimensional surfaces but within complex three-dimensional ECM structures whose composition, porosity, fiber architecture, and stiffness strongly influence invasive behavior [21,22]. Beyond these biophysical aspects, 3D cell culture systems are increasingly being developed for quantitative high-throughput drug screening [23] and for co-culture invasion models that incorporate tumor–stromal cell interactions [24]. Therefore, there remains a need for experimental models that capture tumor cell invasion not only as simple cell movement but also as a dynamic interaction between tumor cells and the surrounding matrix.

In this study, we describe the Freiburg 3D invasion assay, an agarose-based experimental system for the simultaneous analysis of tumor cell migration, tumor cell invasion, and extracellular matrix remodeling. The system consists of two agarose components generated using silicone casting molds: the Gel carrier and the parking pockets. The Gel carrier has defined microcavities with a common loading channel through which tumor cells and subsequently ECM or collagen gel are introduced sequentially. The parking pockets serve as separate support structures that stabilize the cell- or ECM-containing agarose strips from the Gel carrier during culture, handling, and microscopic imaging. Thus, the system enables the standardized spatial combination of tumor cells and ECM/collagen within a defined three-dimensional compartment.

Pijuan et al. described 2D wound-healing, cell-tracking, and 3D Transwell invasion assays as established in vitro assays that are relatively easy to perform, reproducible, rapid, cost-effective, and, importantly, do not require specialized equipment [25]. These features also apply to a large extent to the Freiburg 3D invasion assay. In addition, both adherent and non-adherent cells can be used in our assay, including cell types that are unable to invade through a porous membrane in a Transwell assay. Transwell assays also capture the spatial expansion of tumor cells within a three-dimensional matrix only to a limited extent [26]. The assay presented here differs from these approaches because tumor cells do not migrate through a planar membrane but instead invade from a defined cell aggregate into a three-dimensional ECM or collagen compartment. This allows both invasive protrusions and the cleared matrix area to be followed microscopically and quantified.

Published 3D spheroid invasion assays represent an important advance over 2D and Transwell systems because they enable the invasive outgrowth of tumor cells from compact cell aggregates into a surrounding matrix. Berens et al. described a spheroid invasion assay in which spheroids are first generated by hanging-drop culture and then embedded in a 3D matrix [27]. Matrigel drop-based 3D invasion assays have also been established to quantify the migration and invasion capacity of cancer cells in a three-dimensional matrix [8]. These approaches are more physiologically relevant than simple 2D assays; however, they may be affected by variability in spheroid size, positioning, matrix distribution, and image analysis. The Freiburg 3D invasion assay addresses these points by using defined microcavities that provide a reproducible starting position for the cells, as well as a common loading channel that standardizes the sequential introduction of cells and ECM/collagen.

Another relevant comparison is with more recent 3D tumor-tissue invasion models. Puls et al. developed a 3D tumor-tissue invasion model that supports the reproducible establishment of defined tumor and tissue compartments and was described as an alternative to less standardized spheroid invasion models [28]. Similarly, the Freiburg 3D invasion assay aims to create defined starting conditions for tumor cells and ECM. However, the technical implementation differs as follows: whereas many 3D models rely on embedded spheroids, hydrogels, microfluidics, or bioprinted systems, the assay described here uses an easily fabricated 2.8% agarose-based carrier structure with microcavities and separate parking pockets. This makes the system relatively inexpensive, easy to handle, and accessible to routine laboratories without specialized microfluidic or bioprinting equipment.

In recent years, more complex 3D models, such as tumor-on-chip, bioprinting-based, and dynamic culture models, have been developed. Dogan et al. showed, for example, that ECM composition and 3D bioprinting-based tumor-on-chip systems can be used to study spheroid invasion in defined matrix environments [29]. Similarly, vascularized organotypic spheroid-on chip models have been developed to incorporate additional microenvironmental features, such as perfusion and vascular organization [30]. Dynamic 3D culture systems under flow conditions can additionally account for physiological aspects such as mechanical stress or nutrient transport. Such models are powerful but often technically more complex and less readily implemented in standard cell culture laboratories. The Freiburg 3D invasion assay is therefore not intended to replace highly complex organ-on-chip models but rather to serve as a practical, reproducible, and flexible tool positioned between simple 2D/Transwell assays and technically demanding 3D microsystems.

A central advantage of the described system is the simultaneous measurement of tumor cell invasion and ECM degradation/remodeling. Proteolytic matrix degradation is an essential component of tumor invasion. Matrix metalloproteinases can degrade numerous ECM components and are involved in tumor growth, tissue remodeling, invasion, and metastasis [31]. ADAM and ADAMTS proteases also contribute to pathological tissue remodeling, migration, and tumor invasion through proteolytic activity and regulation of cellular signaling pathways [32]. Consistent with this, we previously showed that ionizing radiation induces ADAM10 activation, leading to degradation and internalization of VE-cadherin, increased endothelial permeability, and enhanced transendothelial migration of tumor cells; these effects were inhibited by GI254023X [16]. Because the Freiburg 3D invasion assay captures not only invasion distance but also cleared matrix area, it is particularly suitable for research questions in which cell movement and protease-dependent matrix remodeling need to be functionally linked. In this study, we show that the assay reflects biologically plausible differences between tumor cell lines. MDA-MB-231 cells displayed stronger invasion and matrix clearing than MCF7 cells, consistent with the known more invasive phenotype of MDA-MB-231 cells compared with luminal MCF7 cells. In addition, treatment with the ADAM10/17 inhibitor GI254023X and irradiation reduced invasion and ECM remodeling, supporting the applicability of the system for pharmacological and radiation-biological studies.

Friedl et al. described collective invasion as an important process in many solid tumors and emphasized the need for suitable experimental models to distinguish collective from single-cell invasion patterns [33]. The Freiburg 3D invasion assay may be particularly useful for this purpose because both collective outgrowth from cell aggregates and single-cell migration can be monitored microscopically. However, standardized image analysis is required to distinguish these invasion modes reliably. Recently, den Daas et al., introduced in vivo-inspired 3D collagen models that use structural guidance cues to study directed tumor invasion [14]. While these models emphasize the role of ECM architecture in guiding cell migration, the Freiburg 3D invasion assay provides a complementary approach by standardizing the spatial positioning of tumor cells and ECM within agarose microcavities. This enables simultaneous longitudinal quantification of invasion distance and ECM clearing in a technically accessible 3D assay.

Despite its advantages, the assay has several limitations. First, the quality of the agarose components depends strongly on precise fabrication. Air bubbles, cracks, incompletely filled microcavities, or irregular agarose structures can affect cell positioning and subsequent ECM loading. Second, manual measurement of the five longest invasion distances and the cleared matrix area remains potentially operator-dependent. Recent work on automated analysis of 3D invasion emphasizes that automated or semi-automated image analysis approaches can improve objectivity and reproducibility of quantification [34]. For future applications, it should therefore be evaluated whether invasion area, maximum invasion distance, mean invasion distance, the number of cells outside the aggregate, and degradation area can be standardized using software-assisted analysis. A further limitation is the transient cooling step required during ECM or collagen loading. Since ECM gel and collagen solutions must be handled cold to prevent premature gelation during pipetting and loading, the cell-containing Gel carriers are briefly cooled before matrix application. Although no pronounced reduction in cell viability was observed under the conditions used here, this step may represent a potential stress factor for sensitive cell types. Therefore, cooling should be kept as short as possible.

An additional limitation concerns the interpretation of matrix degradation. Microscopically visible ECM-cleared/degraded areas provide a functional indication of matrix remodeling but do not by themselves identify the specific proteases involved. For mechanistic studies, the assay should therefore be combined with molecular biological and biochemical methods, such as qPCR, Western blotting, protease activity assays, immunofluorescence, specific inhibition, or genetic knockdown. This is particularly relevant when proteases are investigated as causal mediators of invasion. Combining the Freiburg 3D invasion assay with such molecular readouts could extend the method beyond a purely protocol-based approach toward a mechanistic tumor invasion model. Moreover, fluorescence-based degradation assays using DQ-collagen I, DQ-collagen IV or DQ-gelatin [11], as described by Jedeszko et al., and related DQ-Green BSA approaches [13], such as that described by Goertzen et al., may be used in future applications to validate protease-dependent matrix degradation in parallel with the morphometric ECM-clearing readout of the Freiburg 3D invasion assay.

In summary, the Freiburg 3D invasion assay represents a simple, reproducible, and flexible experimental tool for studying tumor cell invasion and protease-associated ECM remodeling. Compared with 2D wound-healing assays and Transwell systems, it provides a spatially defined 3D matrix environment and longitudinal imaging. Compared with classical spheroid invasion assays, it improves standardization of the starting position by using microcavities and a common loading channel. Compared with complex tumor-on-chip or bioprinting systems, it is technically less demanding and therefore easier to implement in standard laboratories. The method is particularly suitable for comparative analyses of different tumor cell lines, ECM compositions, and therapeutic interventions. Future work should further develop the method through automated image analysis, direct protease activity measurements, and expanded molecular validation, thereby strengthening its use as a mechanistic platform for tumor invasion research.

## Figures and Tables

**Table 2:** Troubleshooting guide for the Freiburg 3D invasion assay.

Step	Problem	Possible Reason	Solution
1	Air bubbles in the cavities 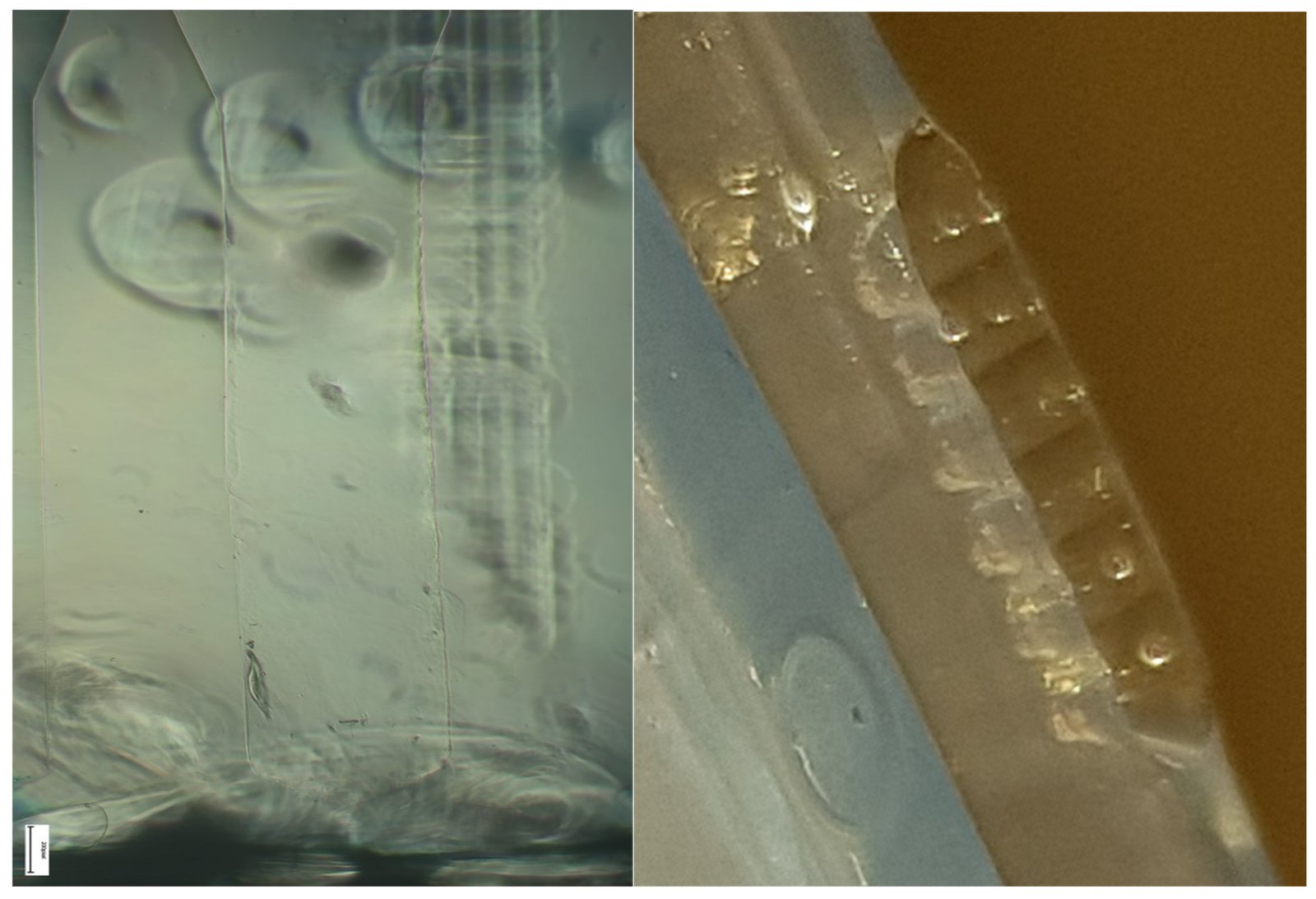	Air bubbles may form during casting of the agarose solution into the molds or during filling of the channel with ECM.	Use a spatula to gently rub the regions containing pockets or cavities to remove air bubbles.
1	Cracks or breaks 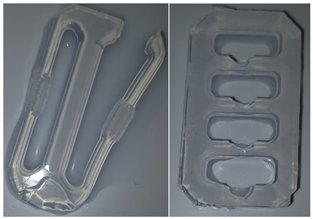	Excessive stretching of the molds or uneven casting of the agarose solution.	Carefully release the agarose gel from the mold by applying even pressure. Patience and manual precision are required during removal. Ensure complete filling of the molds during casting.
1	Irregular columns highlighted by red line 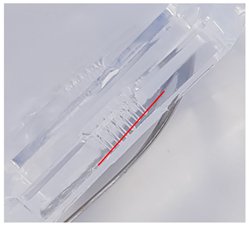	The agarose solution is no longer sufficiently liquid at the time of casting.	Ensure that the agarose solution remains on the magnetic stirrer or heating plate and is stirred continuously.
1	Artifacts 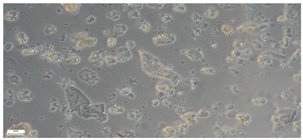	The agarose solution is not fully dissolved or sufficiently mixed.	Dissolve the agarose solution completely in the microwave and mix thoroughly. Ensure that the agarose powder and distilled water are clean.
1	Contamination by mycoplasma, bacteria, or fungi 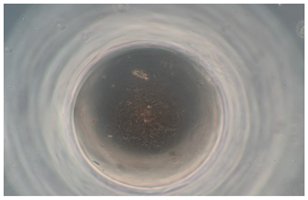	Insufficient sterilization time.	If a different UV source is used, adjust the irradiation time accordingly.
2	Air bubbles during ECM loading into the channel 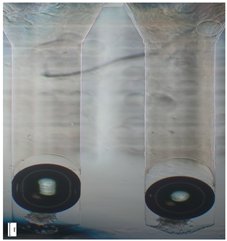	The channel was dried completely before ECM loading.	Do not dry the channel completely before filling it with ECM. Otherwise, the ECM solution does not descend evenly into the cavities.

## Data Availability

The data supporting the findings of this study are available from the corresponding author upon reasonable request.

## Supplementary Materials

The following supporting information can be downloaded at: https://www.mdpi.com/article/10.3390/mps9040112/s1, Figure S1: Technical schematics of silicone molds and matrix; Figure S2: Technical drawings of master molds for silicone casting molds; Figure S3: Cell growth and cell invasion in different matrix conditions. MDA-MB-231 cells (top) and U-251 MG cells (bottom) were seeded into the common loading channel of the gel carrier. The channels were either filled with cells alone or with cells followed by collagen type I or ECM gel. The same loading conditions were used for both cell lines and matrix conditions: 6000 cells per channel, corresponding to ca. 1000 cells per cavity followed by 15 µL collagen type I or ECM gel. Both matrix components were kept on ice before being filled into the channel. The same centrifugation condition was applied in all cases (300 g, 2 min). The gel carriers were then incubated at 37 °C and 5% CO_2_ and images were acquired on day 1, day 6 and day 10. (Scale Bar = 0.5 mm); Figure S4: Cell viabilty. (A,B) Clonogenic survival of (A) MDA-MB-231 and (B) MCF7 cells after treatment with 10 µM GI254023X and irradiation with 0, 2, 4 or 8 Gy. For each condition, 150 cells were seeded, stained with crystal violet after approximately 12 to 14 days and the number of colonies with more than 50 cells were counted. (C,D) Proliferation of (C) MDA-MB-231 and (D) MCF7 cells after treatmemt with different concentrations of GI254023X. Cells were seeded in microtiter plates at 5000 cells per well and treated with 0 (DMSO control), 6.25, 12.5, 25, 50 or 100 µM GI254023X. After 24, 48 and 72 h metabolic activity was determined colorimetrically using MTT assay.

## Abbreviations

The following abbreviations are used in this manuscript:

2D	two-dimensional
3D	three-dimensional
DMEM	Dulbecco’s Modified Eagle Medium
DPBS	Dulbecco’s Phosphate-buffered Saline
ECM	Extracellular Matrix
EMT	Epithelial–Mesenchymal Transition
MMP	Metalloproteinase
FCS	Fetal Calf Serum
P/S	Penicillin/Streptomycin
PTFE	Polytetrafluoroethylene
RPMI	Roswell Park Memorial Institute Medium
uPA	urokinas-type Plasmonogen Activator
