# Supplementary material for: Agarose-Based 3D Invasion Assay for Simultaneous Quantification of Tumor Cell Invasion and Extracellular Matrix Degradation
Source: Methods Protoc. 2026 Jul 28;9(4):112. doi: 10.3390/mps9040112 (PMC13515452; doi:10.3390/mps9040112)
Supplement: Supplementary file 1 [file mps-09-00112-s001.zip › Figure S1_Technical schematics of silicone molds and matrix.pdf]

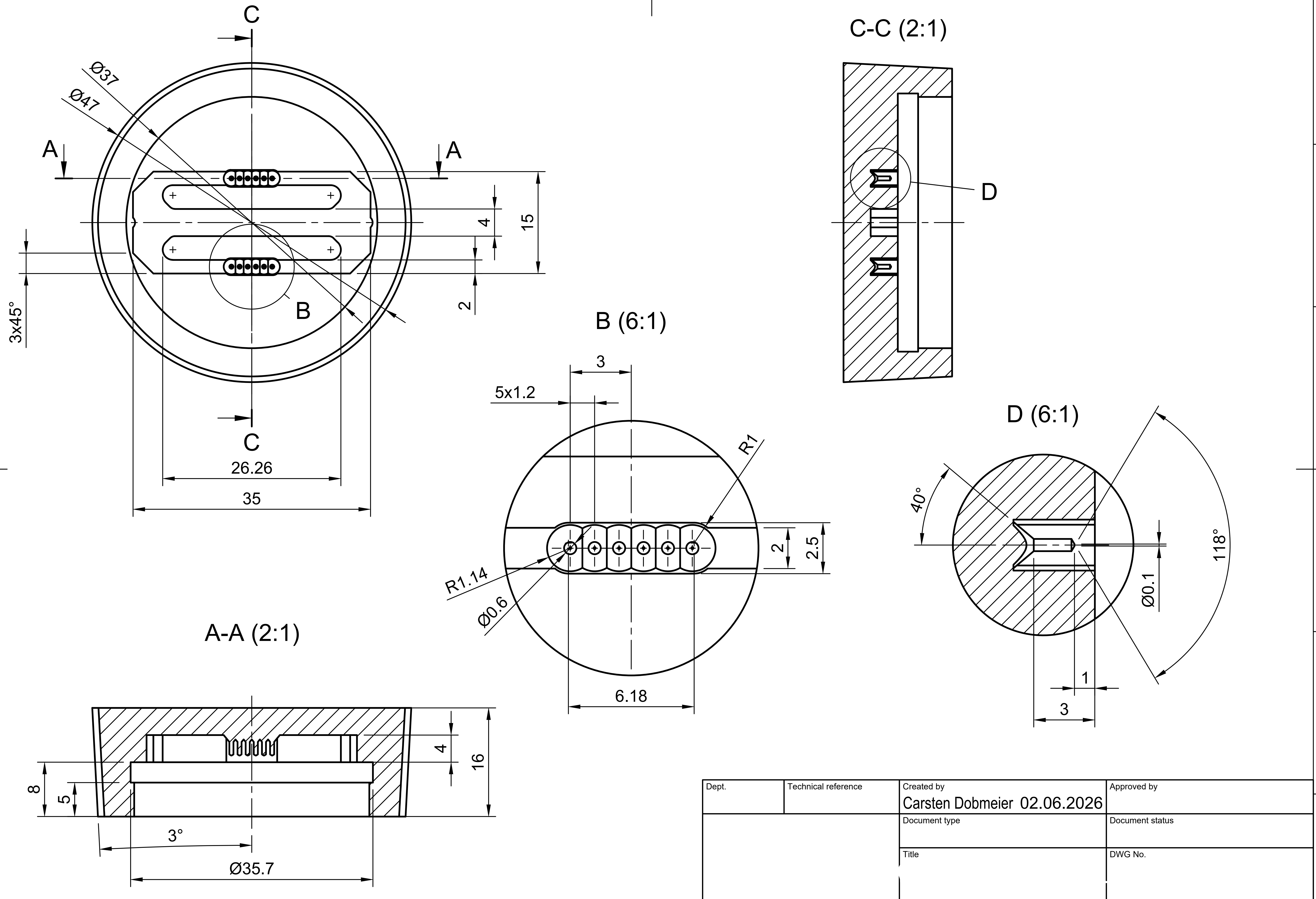

Dept.	Technical reference	Created by	Approved by
		Carsten Dobmeier 02.06.2026	
		Document type	Document status
		Title	DWG No.
		Silicone mold / Gel carrier	Rev. / Date of issue / Sheet 1/2

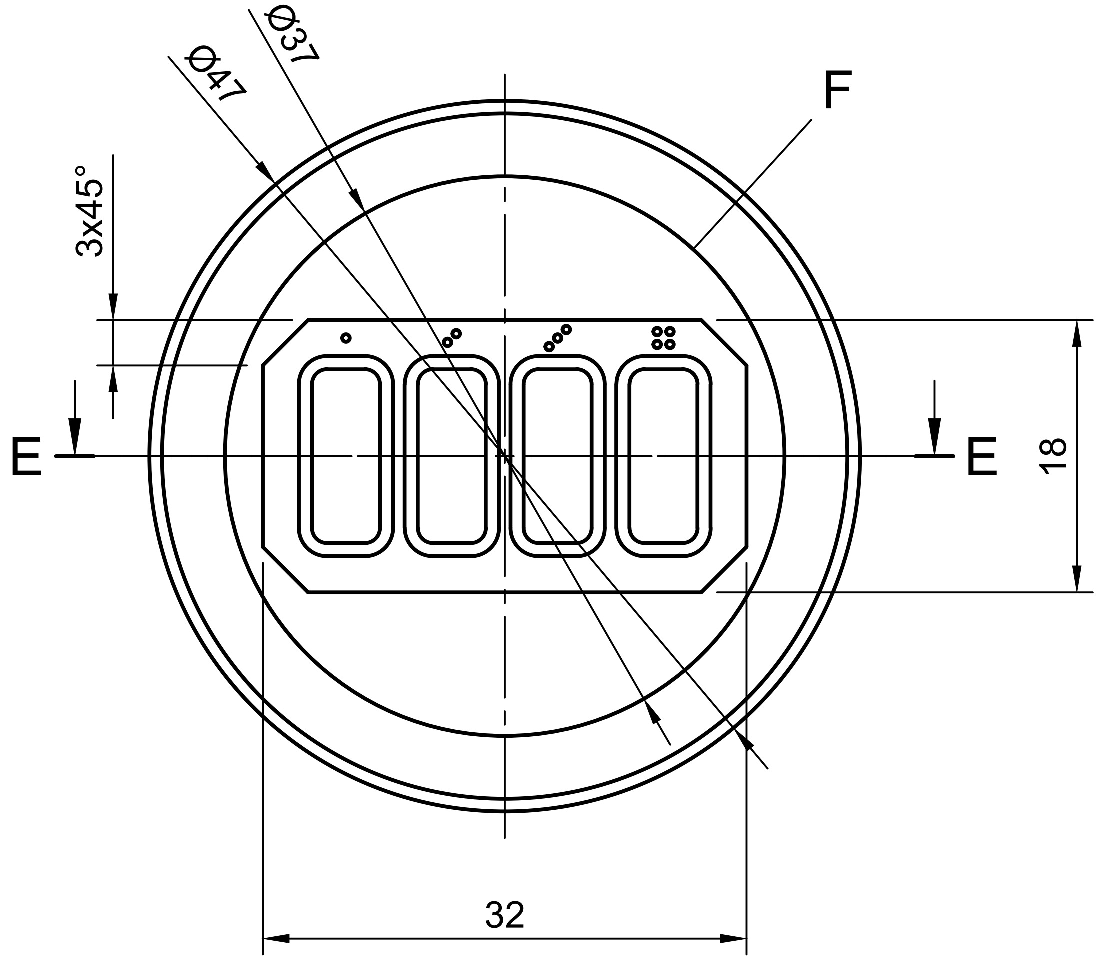

F (4:1)

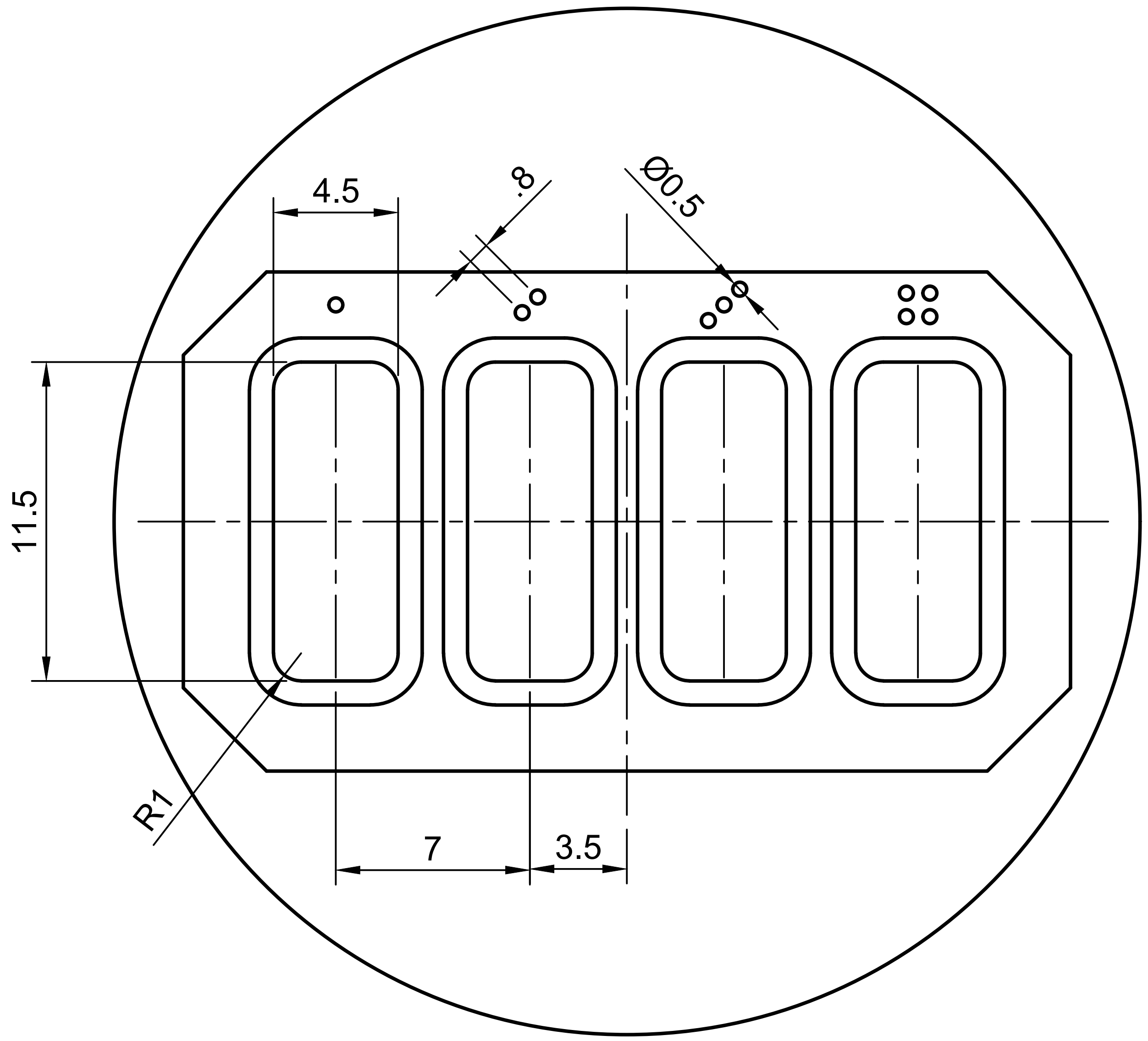

E-E (2:1)

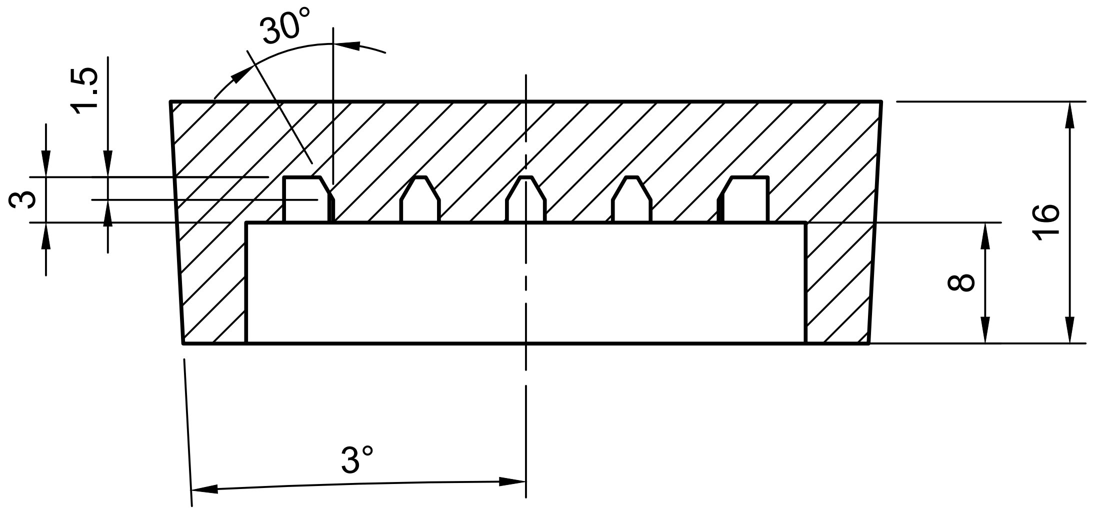

Dept.	Technical reference	Created by	Approved by
		Carsten Dobmeier 02.06.2026	
		Document type	Document status
		Title	DWG No.
		Silicone mold / Pocket	Rev. / Date of issue / Sheet 2/2
