# Supplementary material for: Agarose-Based 3D Invasion Assay for Simultaneous Quantification of Tumor Cell Invasion and Extracellular Matrix Degradation
Source: Methods Protoc. 2026 Jul 28;9(4):112. doi: 10.3390/mps9040112 (PMC13515452; doi:10.3390/mps9040112)
Supplement: Supplementary file 1 [file mps-09-00112-s001.zip › Figure S2_Technical drawings of master molds for silicone casting molds.pdf]

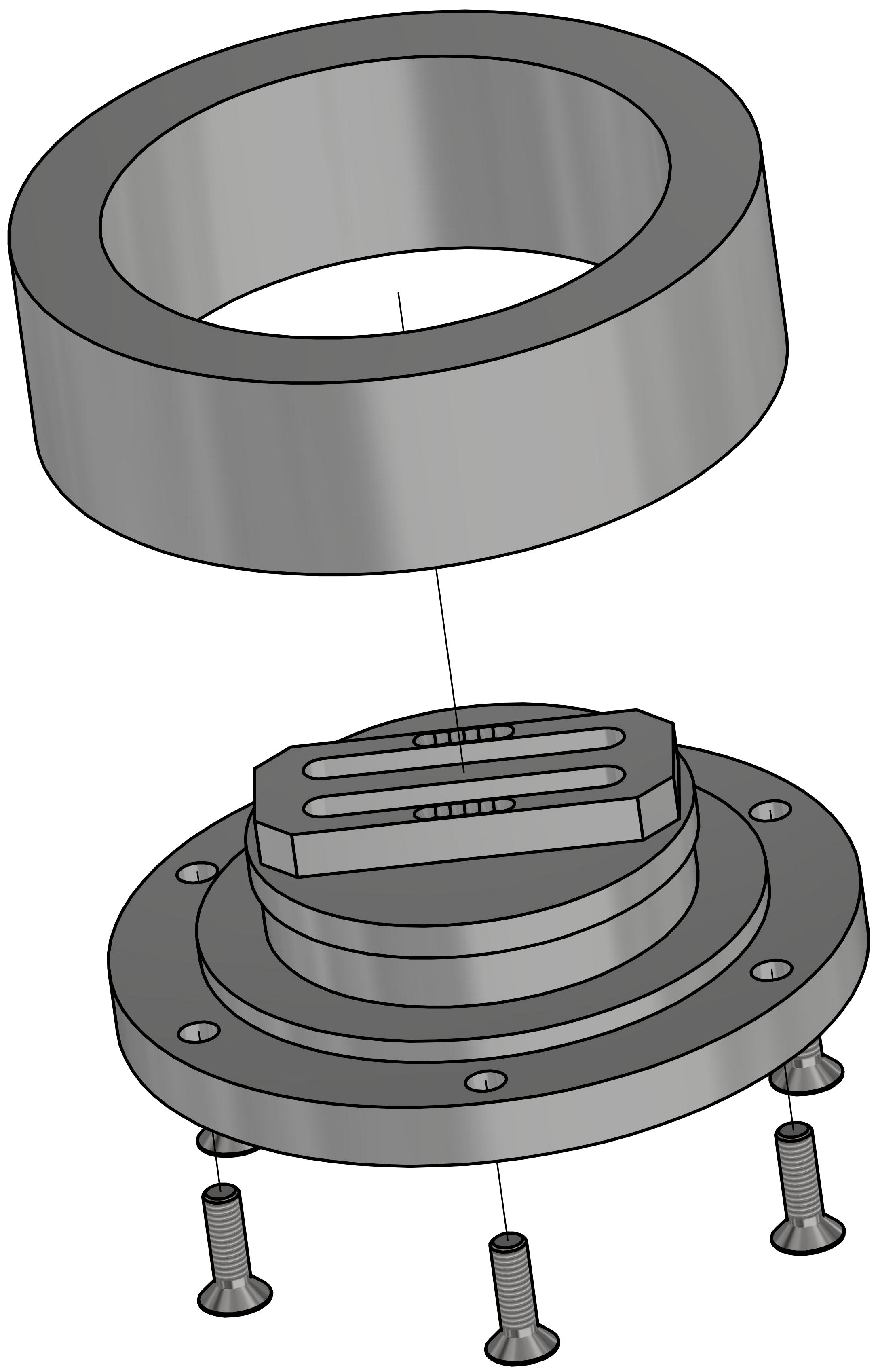

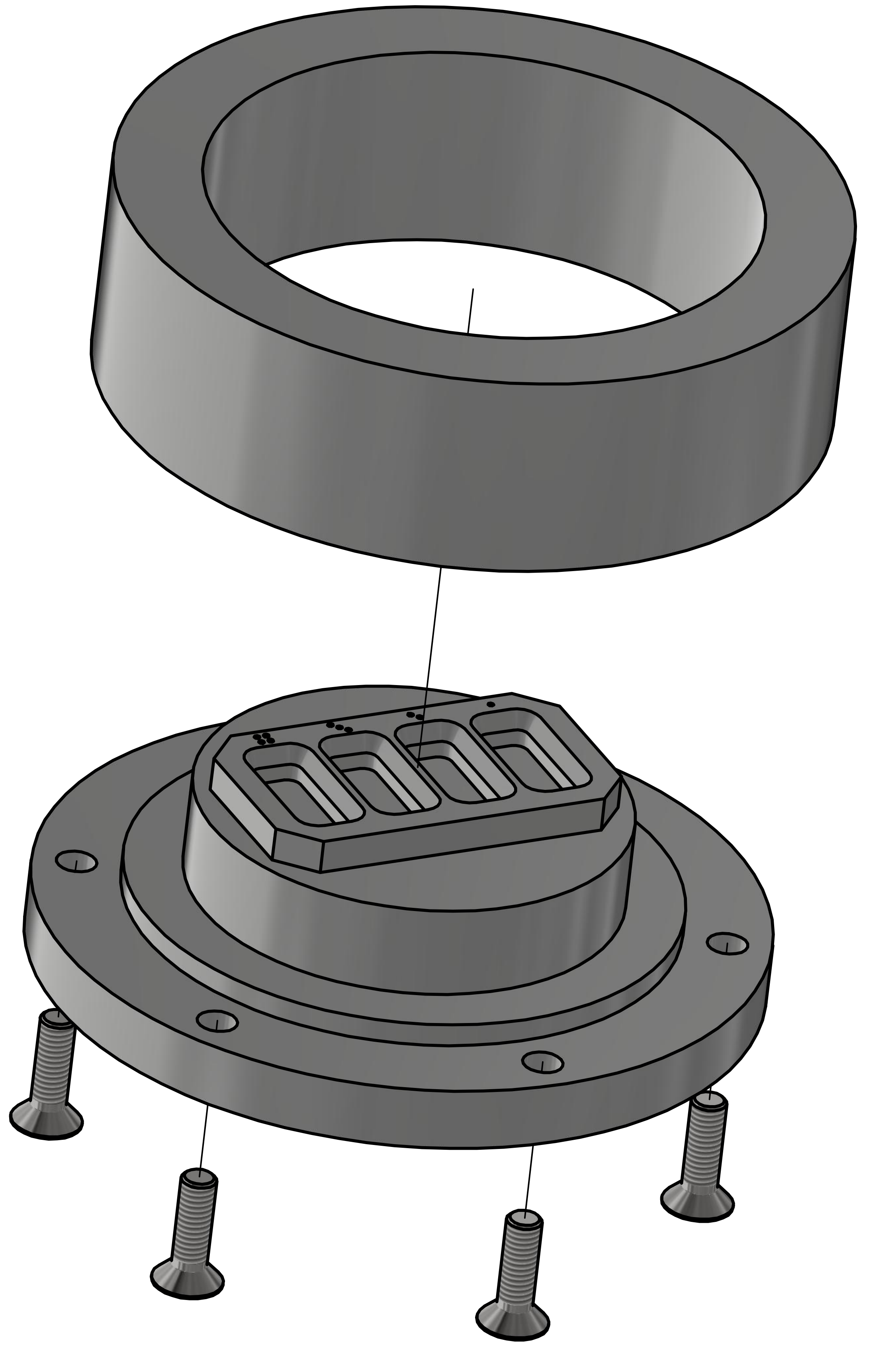

Name<br>Lehrstuhl	Allgemein-<br>toleranz<br>ISO 2768-f			Maßstab:	Gewicht:0,109 kg
Chemie und Biochemie		Datum	Name	PTFE-Formen	
	Gezeichnet	08.07.2026	B. Alban		
	Kontrolliert				
	Norm				
	Fertigungsfreigabe: Nein				1<br>A2
Status | Änderungen | Datum | Name					

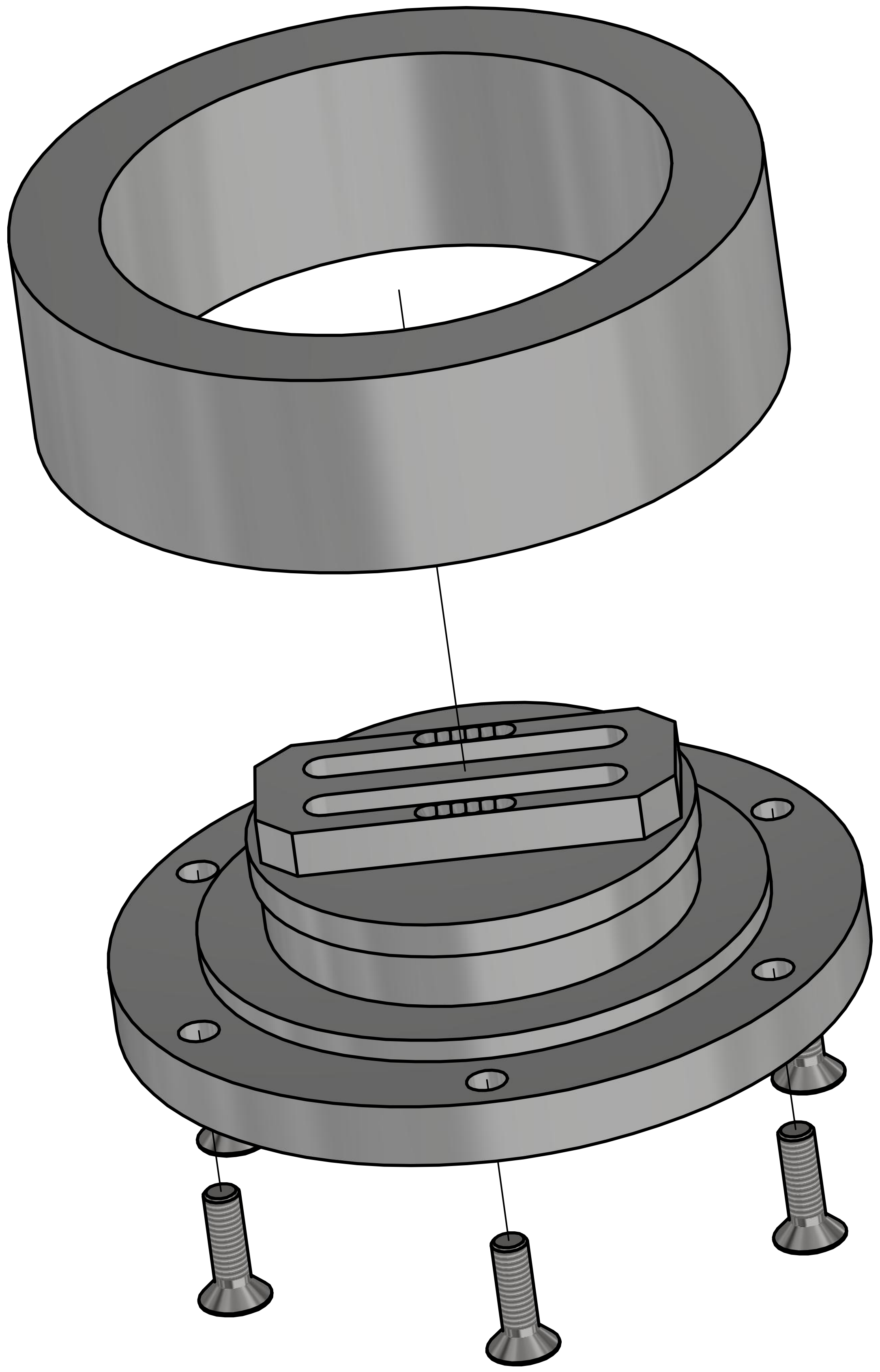

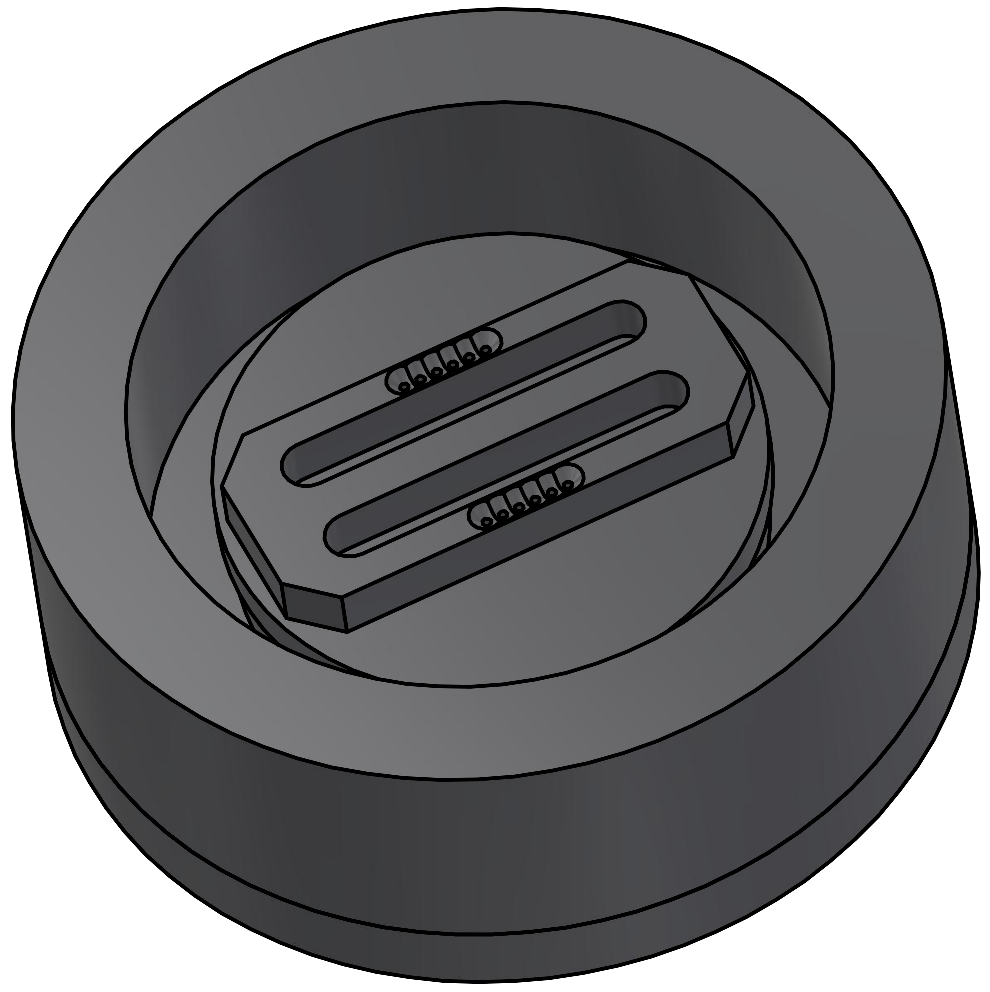

Name<br>Lehrstuhl	Allgemein-<br>toleranz<br>ISO 2768-f	Maßstab:	Gewicht:0,109 kg
Chemie und Biochemie	Datum / Name<br>Gezeichnet 08.07.2026 B. Alban<br>Kontrolliert<br>Norm	Matrize-Zusammenbau	
Status / Anderungen / Datum / Name	Fertigungsfreigabe: Nein		2<br>A2

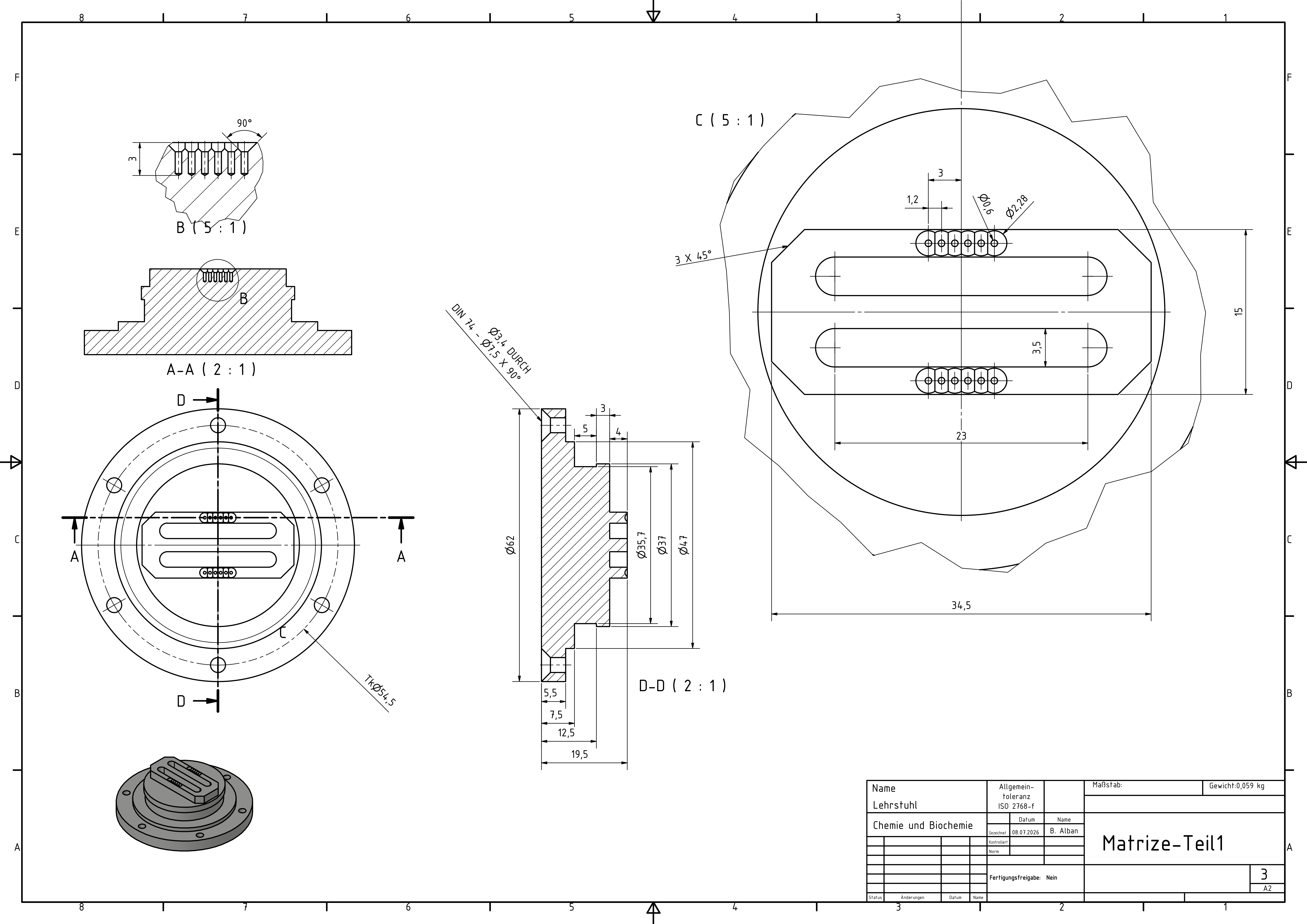
90°
3
B (5 : 1)
B
A-A (2 : 1)
D
A
A
D
C
TkØ54,5
DIN 74 - Ø3,4 DURCH
- Ø7,5 X 90°
3
5
4
Ø62
Ø35,7
Ø37
Ø47
5,5
7,5
12,5
19,5
D-D (2 : 1)
C (5 : 1)
3
1,2
Ø0,6
Ø2,28
3 X 45°
15
3,5
23
34,5
Name
Lehrstuhl
Chemie und Biochemie
Allgemein-
toleranz
ISO 2768-f
Datum
Name
Gezeichnet
08.07.2026
B. Alban
Kontrolliert
Norm
Fertigungsfreigabe: Nein
Status
Anderungen
Datum
Name
Maßstab:
Gewicht:0,059 kg
Matrize-Teil1
3
A2

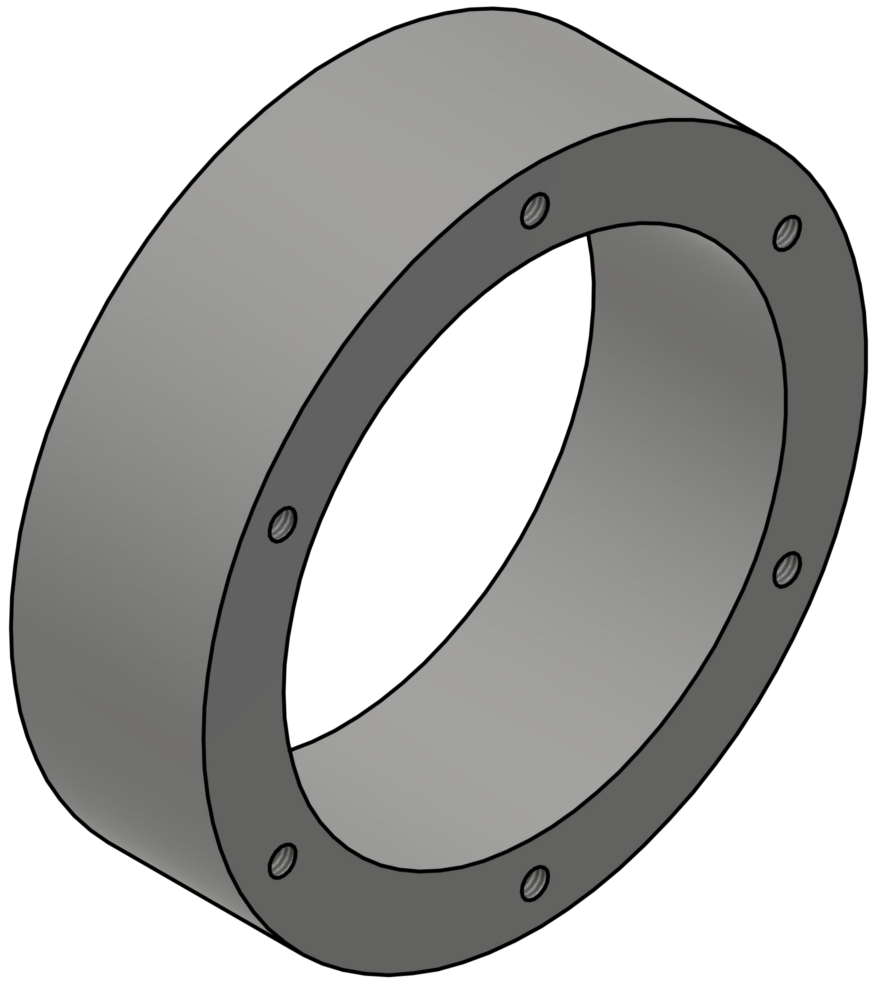

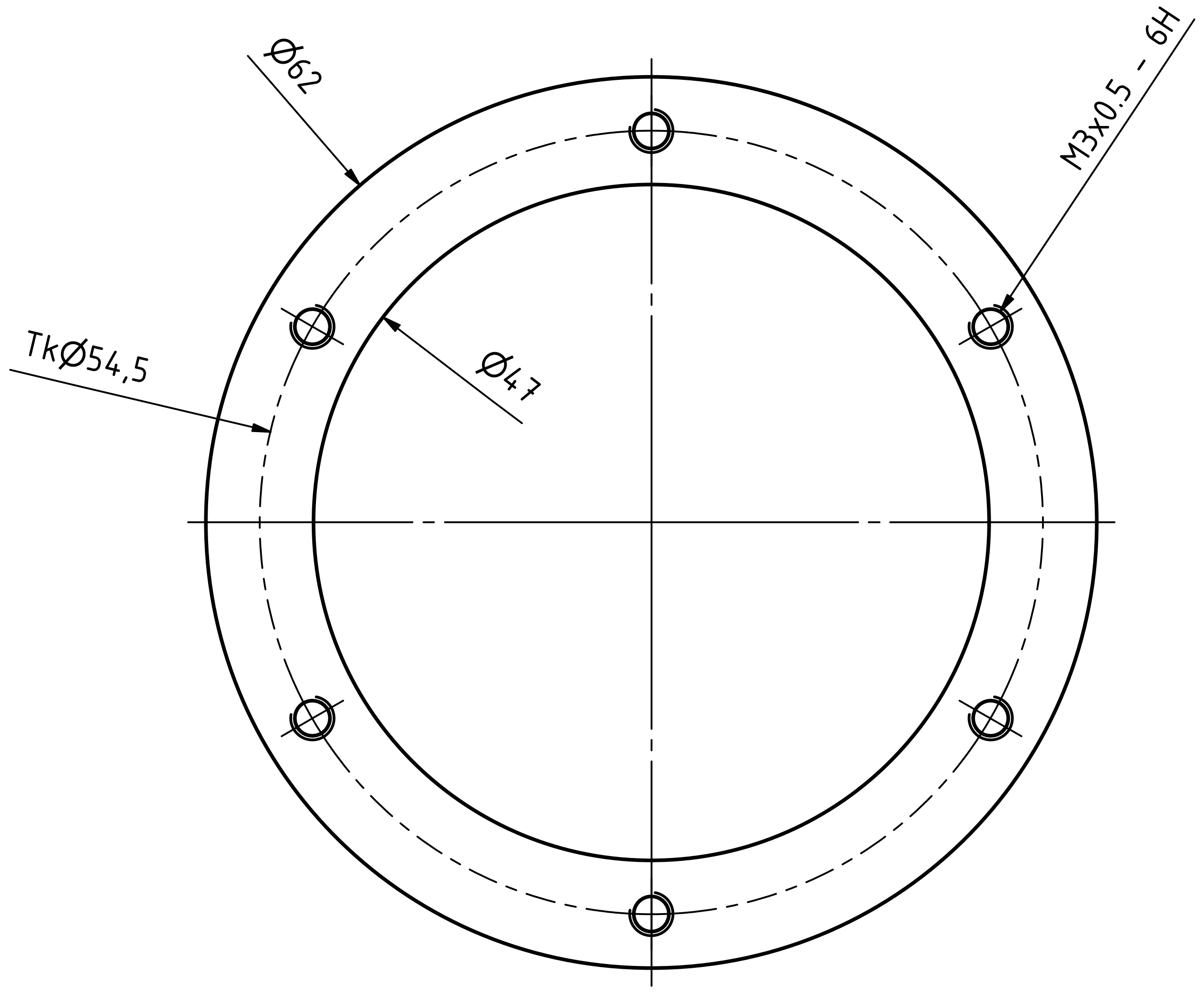

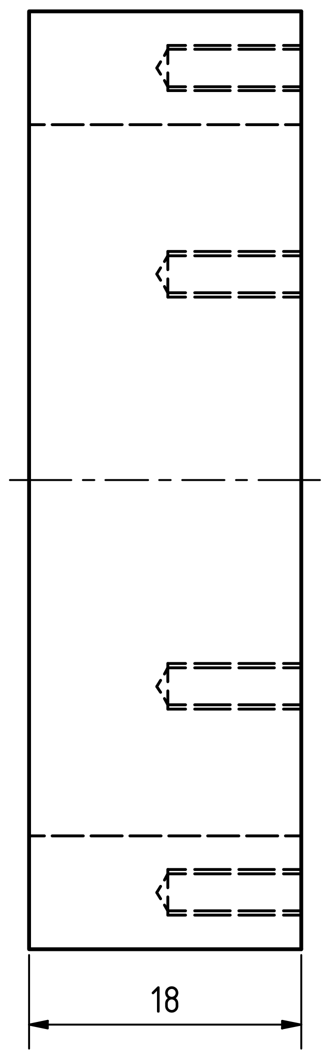

Name<br>Lehrstuhl	Allgemein-<br>toleranz<br>ISO 2768-f		Maßstab:	Gewicht:0,046 kg
Chemie und Biochemie		Datum / Name	Matrize-Teil2	
	Gezeichnet	08.07.2026 / B. Alban		
	Kontrolliert			
	Norm			
	Fertigungsfreigabe: Nein			4<br>A2
Status / Anderungen / Datum / Name				

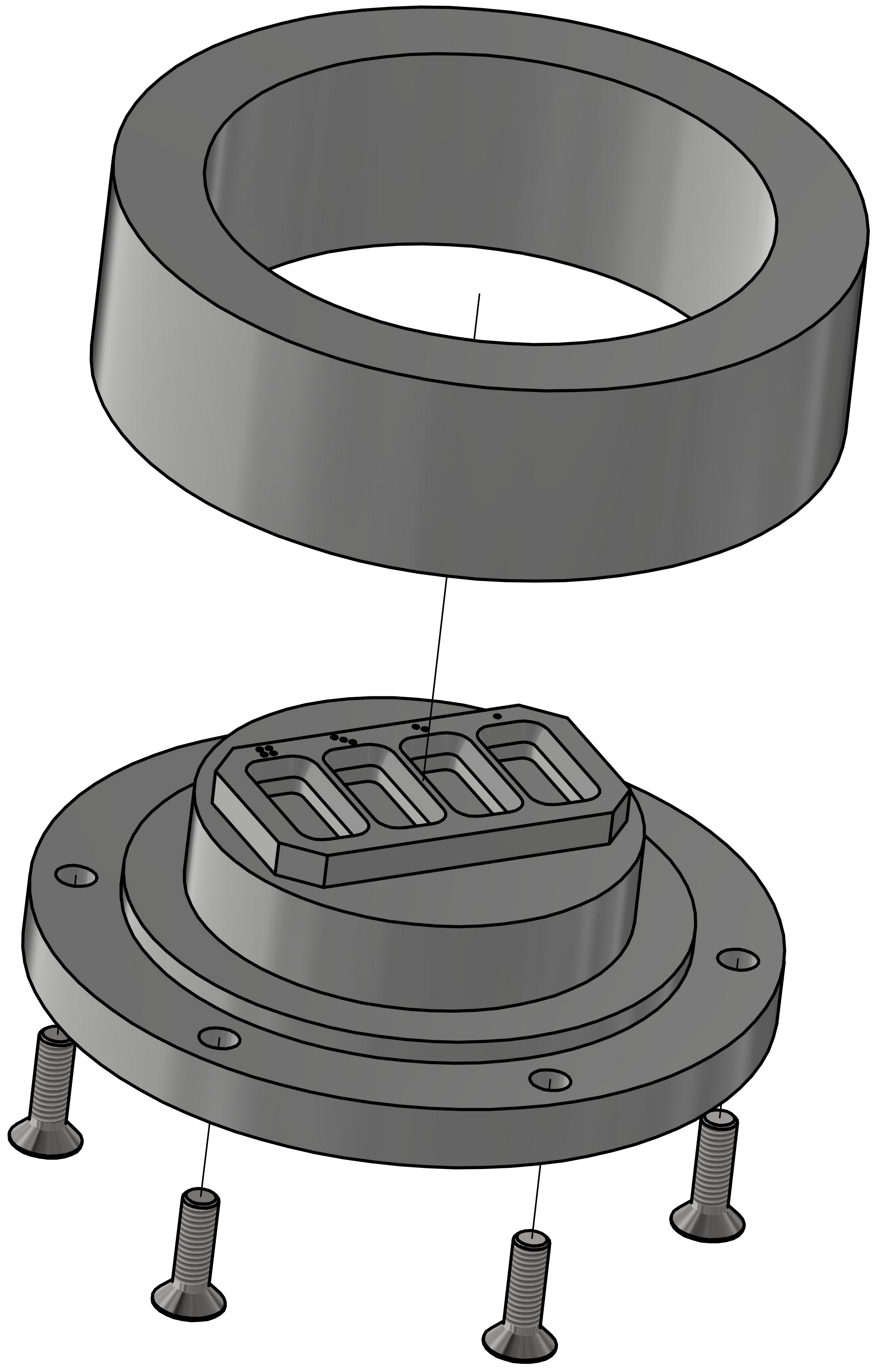

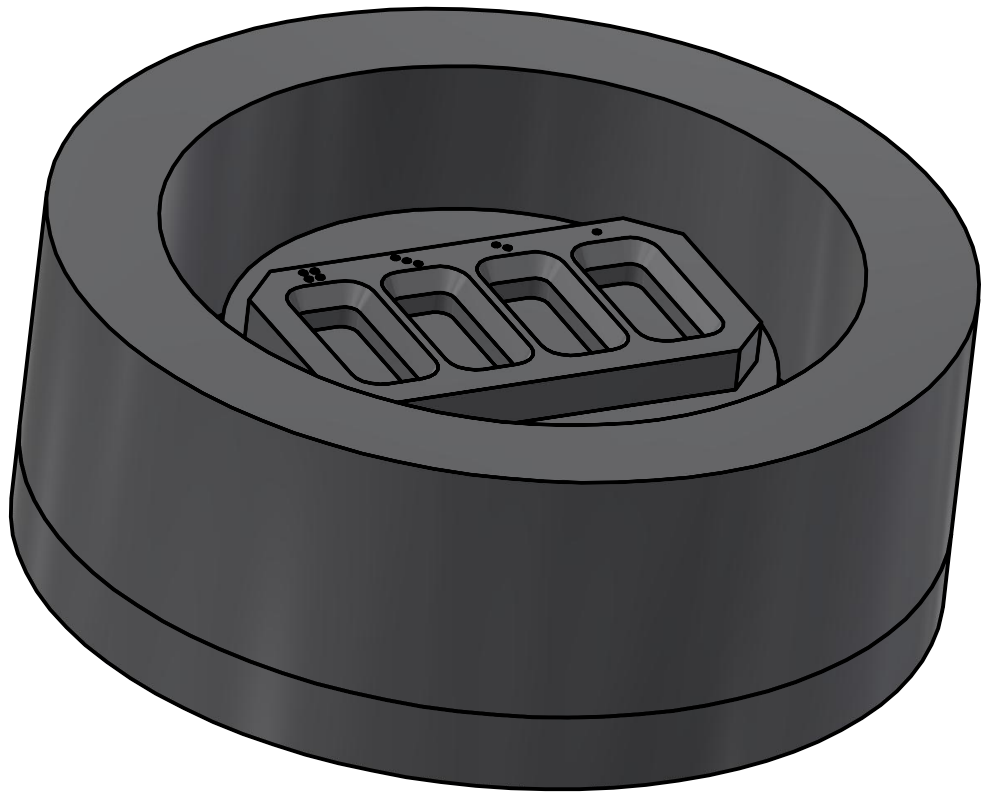

Name<br>Lehrstuhl	Allgemein-<br>toleranz<br>ISO 2768-f	Maßstab:	Gewicht:0,109 kg
Chemie und Biochemie	Datum / Name		
	Gezeichnet 08.07.2026 B. Alban	Pocket Zusammenbau	
	Kontrolliert		
	Norm		
	Fertigungsfreigabe: Nein		5
Status / Anderungen / Datum / Name			A2

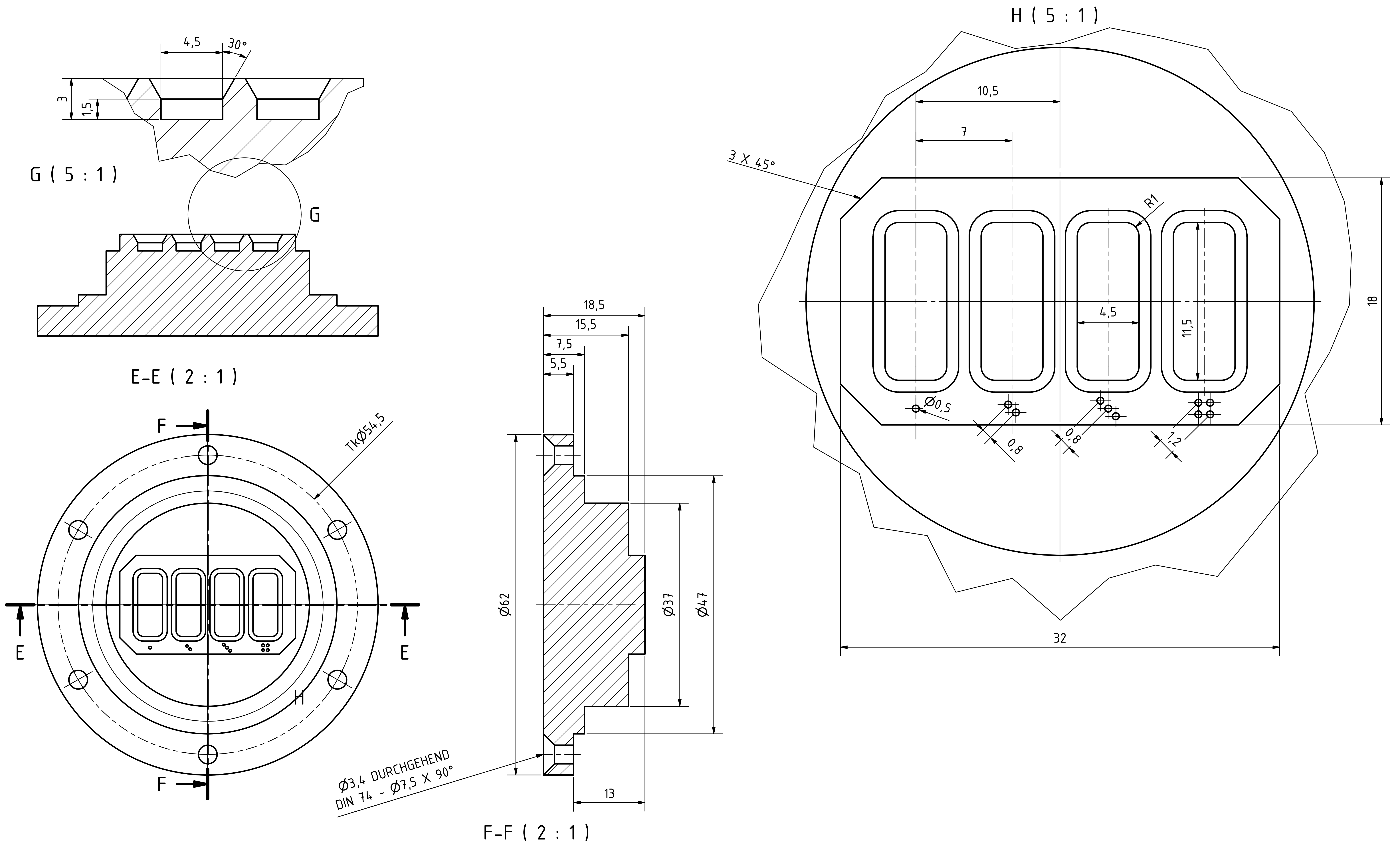

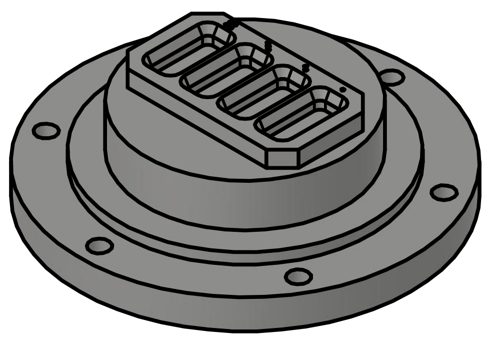

Name	Allgemein-toleranz ISO 2768-f		Maßstab:	Gewicht:0,059 kg
Lehrstuhl				
Chemie und Biochemie		Datum	Name	
	Gezeichnet	08.07.2026	B. Alban	Pocket-Teil1
	Kontrolliert			
	Norm			
	Fertigungsfreigabe: Nein			6
Status / Anderungen / Datum / Name				A2

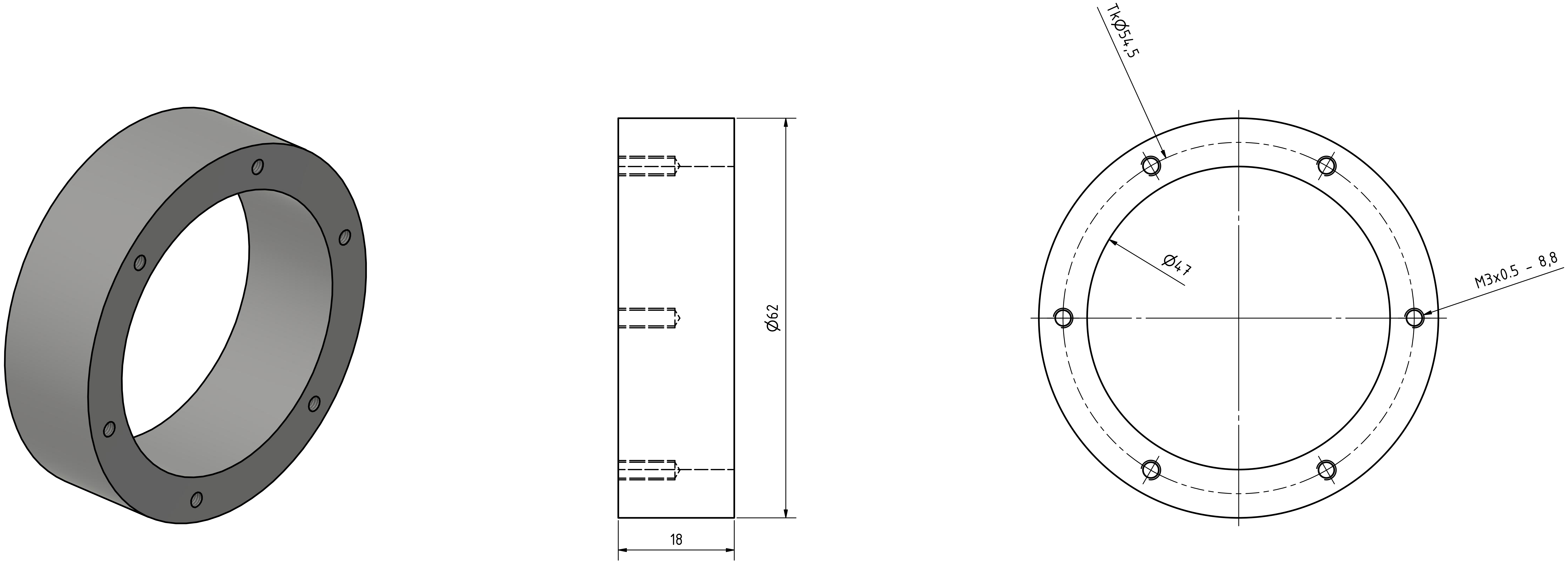

Name<br>Lehrstuhl	Allgemein-<br>toleranz<br>ISO 2768-f			Maßstab:	Gewicht:0,046 kg
Chemie und Biochemie		Datum	Name	Pocket-Teil2	
	Gezeichnet	08.07.2026	B. Alban		
	Kontrolliert				
	Norm				
	Fertigungsfreigabe: Nein				7<br>A2
Status / Anderungen / Datum / Name					
