# Supplementary material for: Agarose-Based 3D Invasion Assay for Simultaneous Quantification of Tumor Cell Invasion and Extracellular Matrix Degradation
Source: Methods Protoc. 2026 Jul 28;9(4):112. doi: 10.3390/mps9040112 (PMC13515452; doi:10.3390/mps9040112)
Supplement: Supplementary file 1 [file mps-09-00112-s001.zip › Figure S3.pdf]

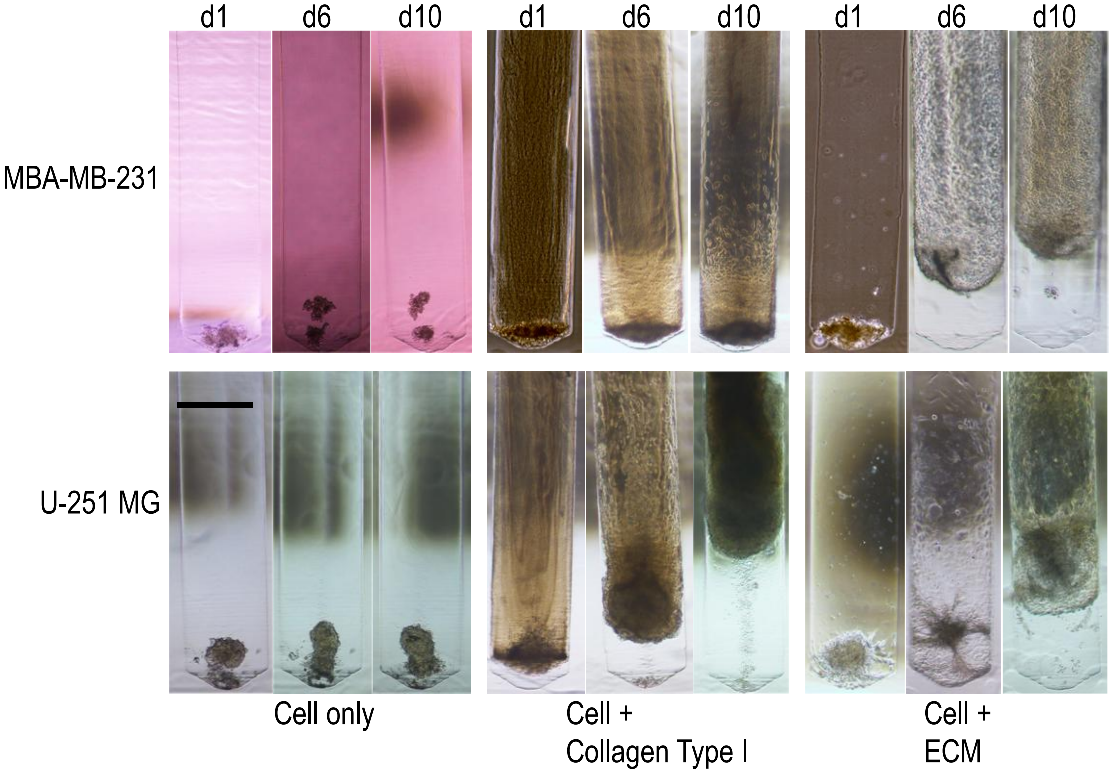

Figure S3. Cell growth and cell invasion in different matrix conditions. MDA-MB-231 cells (top) and U-251 MG cells (bottom) were seeded into the common loading channel of the gel carrier. The channels were either filled with cells alone or with cells followed by collagen type I or ECM gel. The same loading conditions were used for both cell lines and matrix conditions: 6000 cells per channel, corresponding to ca. 1000 cells per cavity followed by 15 µl collagen type I or ECM gel. Both matrix components were kept on ice before being filled into the channel. The same centrifugation condition was applied in all cases (300 g, 2 min). The gel carriers were then incubated at 37°C and 5 % CO_2 and images were acquired on day 1, day 6 and day 10. (Scale Bar = 0.5 mm).
