# Supplementary material for: Agarose-Based 3D Invasion Assay for Simultaneous Quantification of Tumor Cell Invasion and Extracellular Matrix Degradation
Source: Methods Protoc. 2026 Jul 28;9(4):112. doi: 10.3390/mps9040112 (PMC13515452; doi:10.3390/mps9040112)
Supplement: Supplementary file 1 [file mps-09-00112-s001.zip › Figure S4.pdf]

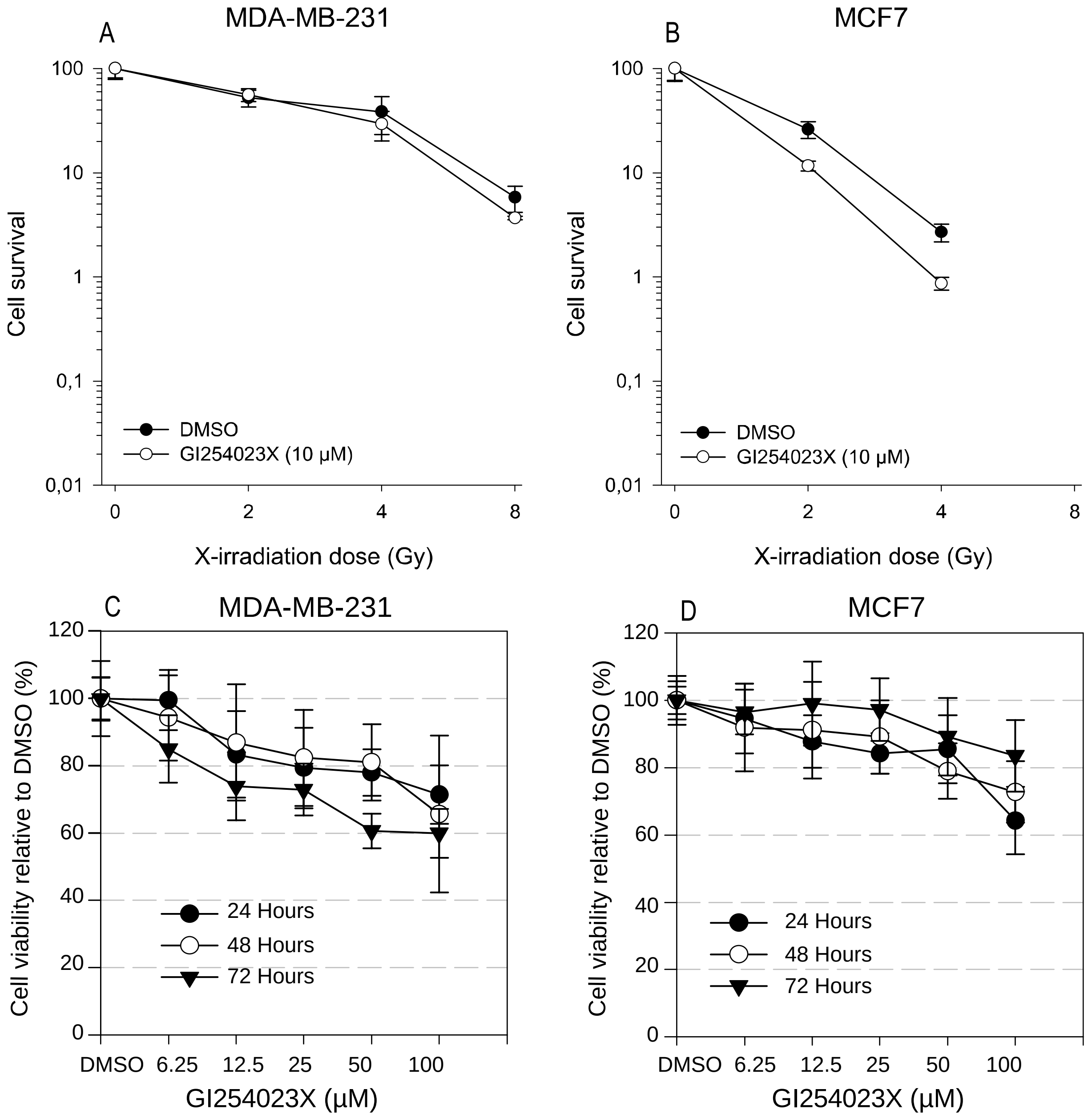


Figure S4. Cell viabilty. (A,B) Clonogenic survival of (A) MDA-MB-231 and (B) MCF7 cells after treatment with 10 µM GI254023X and irradiation with 0, 2, 4 or 8 Gy. For each condition, 150 cells were seeded, stained with crystal violet after approximately 12 to 14 days and the number of colonies with more than 50 cells were counted. (C,D) Proliferation of (C) MDA-MB-231 and (D) MCF7 cells after treatmemt with different concentrations of GI254023X. Cells were seeded in microtiter plates at 5000 cells per well and treated with 0 (DMSO control), 6.25, 12.5, 25, 50 or 100 µM GI254023X. After 24, 48 and 72h metabolic activity was determined colorimetrically using MTT assay.
